# Cryo‐EM and NMR Reveal a Membrane‐Gated Doc2B–Munc13‐1 Complex and a Helical Doc2B‐SNARE‐Binding Interface

**DOI:** 10.1002/advs.78011

**Published:** 2026-11-03

**Authors:** Cyrus T. Rastegar, Julia Powell, Aleksandra Wosztyl, Guillaume David, Klaudia Jaczynska, Xiao‐chen Bai, Yan Han, Zhe Chen, Josep Rizo

**Affiliations:** ^1^ Department of Biophysics University of Texas Southwestern Medical Center Dallas Texas USA; ^2^ Department of Biochemistry University of Texas Southwestern Medical Center Dallas Texas USA; ^3^ Department of Pharmacology University of Texas Southwestern Medical Center Dallas Texas USA

**Keywords:** BindCraft, Doc2, Munc13, neurotransmitter release, SNAREs, synaptic vesicle fusion, synaptotagmin

## Abstract

Doc2s modulate neurotransmitter release through interactions with Munc13‐1, SNAREs, lipids, and Ca^2+^, which involve the Doc2 C_2_ domains and Munc13‐interacting domain (MID). However, it is unclear how these interactions are coupled, how Doc2s bind to Munc13‐1, and how they bind to the SNAREs. Here we determined the cryo‐electron microscopy structure of a Doc2B‐Munc13‐1 complex aided by an AI‐designed Munc13‐1 binder, revealing how Doc2B MID binds to a Munc13‐1 hydrophobic pocket essential for opening syntaxin‐1 and explaining why Doc2B inhibits SNARE assembly. NMR and fluorescence spectroscopy show that Doc2B MID binds lipids and that MID‐lipid and MID‐Munc13‐1 interactions are mutually exclusive, suggesting that membrane binding helps release the inhibition of Munc13‐1. NMR shows that Doc2B binds to the SNARE complex via two helices of C_2_B. Based on these results and liposome fusion assays, we propose a working model whereby: 1. Doc2B MID‐Munc13‐1 interactions facilitate synergistic binding of both proteins to the plasma membrane, with the MID inhibiting Munc13‐1. 2. Membrane binding facilitates MID‐lipid interactions that transiently relieve the inhibition of Munc13‐1, allowing SNARE complex assembly. 3. Doc2B binding to the SNARE complex inhibits neurotransmitter release but generates a primed state that mediates neurotransmitter release by an as‐yet‐unknown mechanism.

## Graphical abstract

Biophysical studies of Doc2B yield a cryo‐electron microscopy structure of its complex with the synaptic vesicle priming factor Munc13‐1 and its binding site to the SNARE complex that mediates synaptic vesicle fusion. These and other results suggest a novel model whereby Doc2 proteins regulate neurotransmitter release through sequential interactions with Munc13‐1, lipids, SNAREs, and calcium.

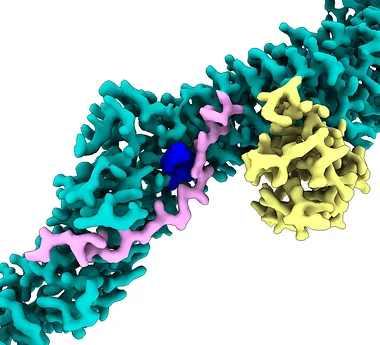

## 1 Introduction

The release of neurotransmitters by Ca^2+^‐evoked synaptic vesicle exocytosis is critical for neuronal communication and involves multiple steps. Synaptic vesicles are tethered to presynaptic active zones, undergo a priming reaction(s) that leaves them ready for release, and fuse with the plasma membrane upon Ca^2+^ influx [1]. Release is controlled by a protein machinery that has been extensively characterized [2, 3, 4], leading to reconstitution of key features of synaptic vesicle fusion with the central components [5, 6, 7] and to definition of their functions. The SNAP receptors (SNAREs) synaptobrevin, syntaxin‐1 and SNAP‐25 form a tight four‐helix bundle called the SNARE complex [8, 9] that mediates membrane fusion [10] by bringing the membranes together [11] and likely acting as a local detergent [12]. This complex is disassembled by N‐ethylmaleimide sensitive factor (NSF) and soluble NSF attachment proteins (SNAPs, no relation to SNAP‐25) after fusion to recycle the SNAREs [8, 13]. Munc18‐1 and Munc13‐1 organize SNARE complex assembly in an NSF‐SNAP‐resistant manner [5, 14] via a pathway that starts with Munc18‐1 bound to a self‐inhibited ‘closed’ conformation of syntaxin‐1 in which the N‐terminal H_abc_ domain [15] binds to the SNARE motif involved in forming the SNARE complex [16, 17] (see domain diagram of syntaxin‐1 in Figure 1A). Munc18‐1 also binds to synaptobrevin, forming a template for SNARE assembly [18, 19, 20] while Munc13‐1 opens syntaxin‐1 [21, 22] and bridges the two membranes [23, 24]. The resulting trans‐SNARE complex binds to Synaptotagmin‐1 (Syt1) [25] and complexin [26], forming a spring‐loaded primed state [27] that is ready for fast (< 1ms), synchronous membrane fusion and neurotransmitter release when Ca^2+^ binds to the two C_2_ domains of Syt1 [28, 29, 30].

**FIGURE 1:**
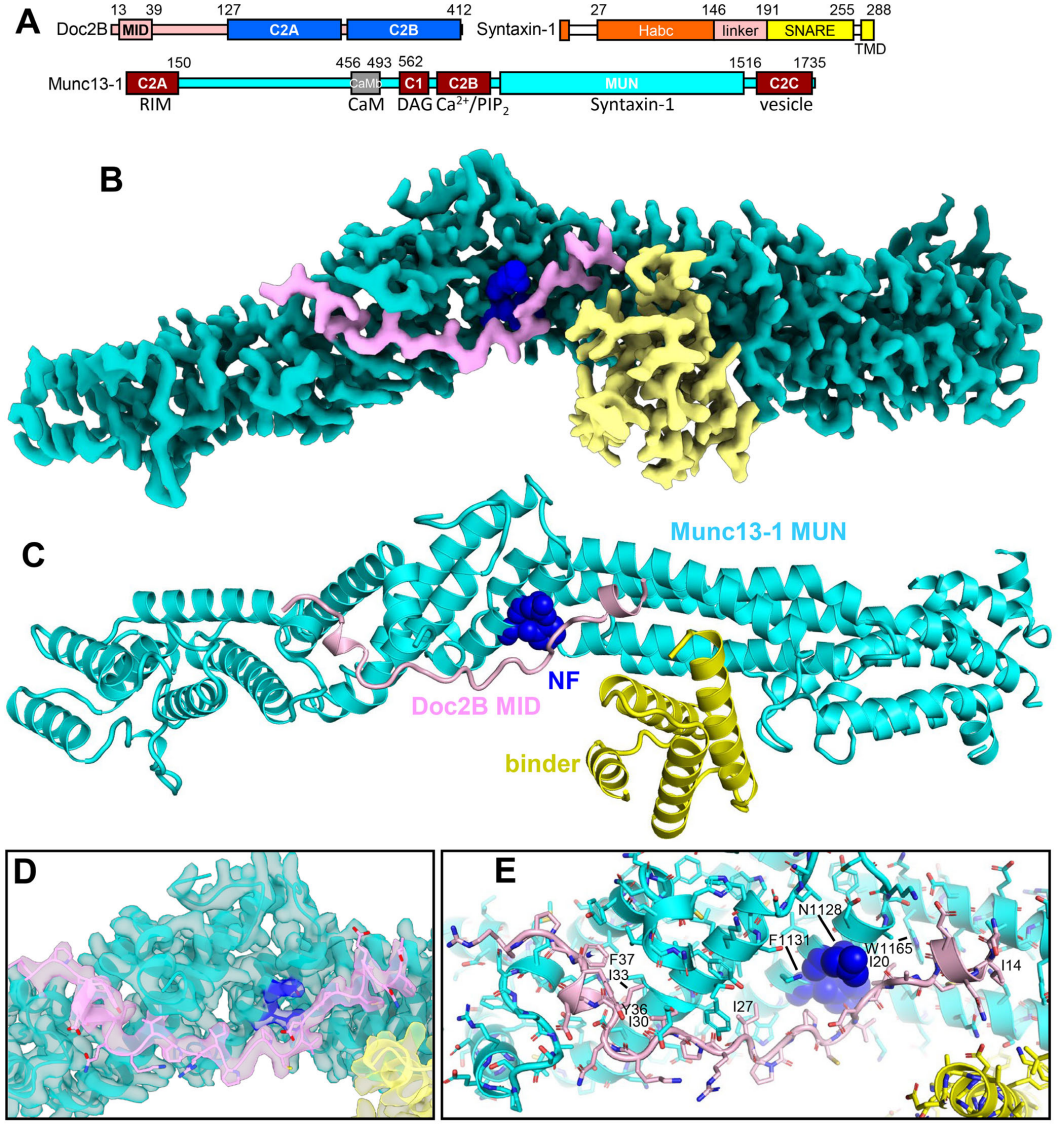
Cryo‐EM structure of the Munc13‐1‐Doc2B complex. (*A*) Domain diagrams of Doc2B, syntaxin‐1, and Munc13‐1. Selected residue numbers are indicated above the diagrams and the different domains are labeled (SNARE = SNARE motif; CaMb = calmodulin binding domain). The binding targets of the Munc13‐1 domains are indicated below the diagram. (*B*) Cryo‐EM map of the Munc13‐1 C_1_C_2_BMUNC_2_C‐Doc2B complex. Note that the C_1_‐C_2_B region and the C_2_C domain present at opposite ends of the MUN domain were not modeled in the 3D reconstruction because motions caused poor resolution in these regions of the map (Figure S4B). In the map, the binder is colored in yellow, Doc2B in pink, and Munc13‐1 in cyan except for the N1128 and F1131 (NF motif), which are in blue. (*C*) Ribbon diagram of the cryo‐EM structure of the Munc13‐1 C_1_C_2_BMUNC_2_C‐Doc2B complex. (*D*) Close‐up view of the region of the cryo‐EM map where the Doc2B MID binds to Munc13‐1 C_1_C_2_BMUNC_2_C superimposed with a ribbon and stick diagram of the 3D model (PDB Entry 37IC). (*E*) Close‐up of the structure of the complex showing ribbon and stick models of the proteins. Selected residues of the interface that were mutated in this or previous studies (see text) are labeled. Nitrogen atoms are blue, oxygen atoms red, sulfur atoms are light orange, and carbon atoms are yellow (binder), pink (Doc2B), or cyan (Munc13‐1).

Neurotransmitter release is also triggered by Ca^2+^ in a slower, asynchronous time scale, can also occur spontaneously and is regulated in a wide variety of presynaptic plasticity processes that alter the release probability, thus underlying many forms of information processing in the brain [31, 32, 33]. Various Ca^2+^ sensors in addition to Syt1 are involved in the distinct forms of release and in Ca^2+^‐dependent presynaptic plasticity, including other Syts [34, 35, 36, 37], double C2‐like domain‐containing proteins (Doc2s) [37], and Munc13‐1 [38], which in addition to being essential for exocytosis [39, 40, 41] acts as a master regulator of release in diverse types of plasticity [2, 42, 43]. Doc2s contain tandem C_2_ domains, similar to Syts, but they are not membrane anchored (Figure 1A) and their roles are distinct from those of Syts. Thus, while synchronous release is abolished and spontaneous release is enhanced in Syt1 KO mice [44, 45], double KO of the two major Doc2 isoforms (Doc2A and Doc2B) [46] or knockdown of Doc2s [47, 48] impairs spontaneous release without altering synchronous release (but see [49]). Absence of Doc2s does lead to changes in synchronous release during repetitive stimulation using different paradigms, including impairments in synaptic augmentation [50] and long‐term potentiation [51]. One study also implicated Doc2s in asynchronous release [48], but others did not [34, 47], and studies in chromaffin cells suggested a role for Doc2s in vesicle priming [52] that was not observed in hippocampal neurons [48].

Some of the functions of Doc2s arise because they bind to Munc13‐1 via a Munc13‐interacting domain (MID) located within an N‐terminal intrinsically disordered region (IDR) [53] (Figure 1A) and co‐translocate with Munc13‐1 to the plasma membrane [54]. Indeed, the isolated Doc2 MID inhibits evoked release [55] and phorbol ester‐dependent potentiation of release [56], and the MID is critical for the role of Doc2 in synaptic augmentation, in vesicle priming in chromaffin cells and in plasma membrane co‐translocation with Munc13‐1 [50, 52, 54]. Intriguingly, binding of the Doc2B MID to Munc13‐1 inhibits the ability of Munc13‐1 to open syntaxin‐1 [57], showing that Doc2s play inhibitory and active functions in release, as most components of the release machinery do [2]. However, the structural basis for these findings is unknown.

The Doc2 C_2_ domains are often assumed to have direct roles in synaptic vesicle fusion through similar mechanisms as Syt1, which have been extensively studied [2, 4, 58], but it is important to note that drastic revisions to these mechanisms were suggested by recent data [59] (see also discussion). Doc2B has been better studied than Doc2A, but they are expected to have similar biochemical properties given their high sequence similarity (75%–80%). The Doc2B C_2_ domains bind multiple Ca^2+^ ions [60], and Ca^2+^ induces binding of the C_2_ domains to membranes [46, 48], as observed for Syt1 [2]. A fragment spanning the Doc2B C_2_ domains (C_2_AB) stimulates SNARE‐dependent liposome fusion in the presence of Ca^2+^, similarly to Syt1 C_2_AB but with a submicromolar apparent Ca^2+^ affinity [46] that contrasts with the ca. 10 µm Ca^2+^ concentrations required for Syt1 activation and triggering of synchronous release [28, 61]. Moreover, elevation of intracellular Ca^2+^ induces translocation of Doc2A and Doc2B to the plasma membrane in chromaffin cells with EC50s of 450 and 175 nm, respectively [62]. These findings suggest that Doc2s function as high‐affinity Ca^2+^ sensors, which bodes well for direct roles in spontaneous release dependent on oscillations in intracellular Ca^2+^ concentration, in release triggered by accumulation of Ca^2+^ during repetitive stimulation, and perhaps in asynchronous release.

Ca^2+^ was also shown to stimulate binding of Doc2B to soluble SNARE complexes [46], but it is important to note that soluble SNARE complexes lack the topological constraints of a membrane, leading to artifactual electrostatic interactions with the polybasic regions of C2 domains, as shown recently for Syt1 [63, 64]. In fact, the Syt1 C_2_B domain binds to membrane‐anchored SNARE complexes in the absence of Ca^2+^ via a so‐called primary interface [25] that is crucial for synaptic vesicle priming, and Ca^2+^ actually weakens this interaction [63], inducing a reorientation of C_2_B on the membrane that is critical to trigger neurotransmitter release [65, 66]. However, the primary interface is not conserved in Doc2s. Hence it is unclear whether Doc2s bind to membrane‐anchored SNARE complexes in the absence or presence of Ca^2+^, and whether they compete with Syt1 for SNARE binding.

The importance of understanding the molecular mechanisms underlying the functions of the proteins that control neurotransmitter release in general and of Doc2s in particular is increasingly being emphasized by the vast number of mutations that are being identified in these proteins in patients with a wide variety of neurological disorders. For instance, coding variants in the three SNAREs, Complexin, Syt1, Munc18‐1 and Munc13‐1 are associated with neurodevelopmental disorders [67, 68]. Additionally, intronic variants that promote cryptic exon inclusion and subsequent loss of Munc13‐1 expression are a major risk factor for amyotrophic lateral sclerosis and frontotemporal dementia [69, 70]. Doc2 proteins have been implicated in neurologic and psychiatric disorders, including Lewy body dementia [71], Parkinson's disease [72], and schizophrenia [73]. Doc2 proteins also play an important role in insulin secretion and may have a protective effect against diabetes [74, 75, 76].

In summary, extensive physiological and genetic data demonstrate the importance of Doc2s and their interaction with Munc13‐1 for brain function, and the complexity of the phenotypes observed in their absence most likely depends on interactions of Doc2s with Munc13‐1, the SNARE complex, Ca^2+^ and the membranes. However, understanding these phenotypes and how Doc2 mutations cause disease is hindered by the lack of information on how Doc2s bind to Munc13‐1, on whether and how Doc2s bind to the SNAREs, and on how these interactions are altered by membranes and Ca^2+^.

To address these questions and shed light on the mechanisms underlying Doc2 function, we have used cryo‐electron microscopy (cryo‐EM), NMR spectroscopy and fluorescence resonance energy transfer (FRET). Cryo‐EM aided by a de novo‐designed Munc13 binder reveals the structural basis of how the Doc2B MID binds to Munc13‐1, explaining why the MID inhibits Munc13‐1. NMR and FRET show that the Doc2B MID binds to lipids, which competes with Munc13‐1 binding and may help relieve the inhibition of Munc13‐1 by the MID. Our data also show that Doc2B binds to membrane‐anchored SNARE complex in the absence of Ca^2+^ through a site distinct from Syt1 involving two helices of the C_2_B domain. These results suggest a model whereby a cascade of events involving the distinct interactions of Doc2s with Munc13‐1, lipids, the SNARE complex and Ca^2+^ governs their functions in neurotransmitter release and presynaptic plasticity.

## 2 Results

### 2.1 Elucidation of the Cryo‐EM Structure of the Munc13‐1 Doc2B Complex Aided by a De Novo Munc13‐1 Binder

The conserved C‐terminal section encompassing the C_1_, C_2_B, MUN and C_2_C domains of Munc13‐1 (C_1_C_2_BMUNC_2_C) (Figure 1A) constitutes its executive region, with the MUN domain mediating syntaxin‐1 opening [21, 22] while the C_1_ and C_2_B domains bind to diacylglycerol (DAG) and PIP_2_ on the plasma membrane and the C_2_C domain binds to the vesicle [23, 24]. The MUN domain mediates binding to the Doc2 MID [53]. To investigate the structural basis for binding of Doc2B to Munc13‐1, we used cryo‐EM. In our first attempt, we prepared a sample consisting of full‐length Doc2B (FL Doc2B) and C_1_C_2_BMUNC_2_C (Figure S1). Although we obtained good quality 2D classes (Figure S1B) and a 3D map with a nominal resolution of 3.13 Å (Figure S1B,D), the resolution was not homogeneous (Figure S1B‐C). Focused refinement and 3D classification at the center of the complex (Figure S1C) yielded a 3D map with clear additional density that could be attributed to Doc2B MID, but the poor map quality precluded definitive identification and reliable model building.

There were four major limitations with this first dataset: 1. severe preferred orientation (Figure S1E); 2. dissociation of Doc2B from a large fraction of the particles; 3. continuous motions; and 4. difficulties with particle alignment, as Munc13‐1 C_1_C_2_BMUNC_2_C is highly elongated and only 40 Å in its narrowest dimension. To overcome the latter two issues, we reasoned that a small nanobody‐like protein binder to the MUN domain could constrain the local flexibility and act as a fiducial marker for particle alignment. To this end, we pursued a binder campaign using BindCraft [77] and identified one binder with a k_D_ of ∼300 nm based on isothermal titration calorimetry (ITC) data (Figure S2A). The binder was expected [78] to interact with a region of the MUN domain close to the N1128,F1131 dyad (NF motif) of the Munc13‐1 MUN domain that plays a key role in opening syntaxin‐1 [22]. These predictions allowed us to design a bivalent binder with the Doc2B IDR flexibly linked to the binder with the goal of increasing overall affinity and mitigating Doc2B dissociation during plunge‐freezing. Indeed, the binder‐Doc2B IDR fusion bound to the MUN domain with very high affinity (k_D_ between sub‐nM and single digit nM; Figure S2B). Finally, to prevent preferred orientations, we incorporated Late Embryogenesis Abundant (LEA) proteins, which form a protective barrier at the air‐water interface [79].

Two additional datasets collected on this new sample resulted in a substantially more isotropic map with an overall resolution of 2.94 Å (Figure 1B and Figures S3 and S4). The highest resolution was at the center of the MUN domain containing the Doc2B binding site, and the worst was at the periphery of MUN and densities corresponding to the C_1_, C_2_B, and C_2_C domains (Figure S4B). Refinement revealed a clear binding interface spanning residues 13–39 of Doc2B, which bind in an extended irregular conformation with two helical turns to grooves and pockets along the MUN domain (Figure 1B–E). Binding involves multiple hydrophobic residues of the MID and the MUN domain, including the NF motif of Munc13‐1 that is key for opening syntaxin‐1 [22] (Figure 1E). Hence, this binding mode explains why the Doc2B MID inhibits Munc13‐1‐dependent SNARE complex assembly [57]. Indeed, some of the grooves and pockets of Munc13‐1 that bind to Doc2B are predicted to bind to the syntaxin‐1 linker region to catalyze opening of the closed conformation [80], suggesting that the syntaxin‐1 linker and Doc2B compete for binding to Munc13‐1.

Our cryo‐EM structure also fits well with ITC results showing that a Doc2B fragment spanning the originally defined MID sequence (residues 1–37) [53] bound to the MUN domain with a 1.47 µm K_D_ whereas a slightly longer fragment (residues 13–60) bound with a K_D_ of 0.23 µm, similar to that observed with the full Doc2B IDR [57]. The impairments in MUN domain‐Doc2B binding caused by mutations in several hydrophobic residues [57] that form the interface in our structure (indicated in Figure 1E) support the validity of our cryo‐EM structure. To further support the model, we used ITC to test how binding of FL Doc2B to the Munc13‐1 MUN domain is affected by a mutation in a hydrophobic residue of MID that is buried in a pocket of MUN not mutated previously (F37A) (see Figure 1E). The K_D_s between MUN and WT or FL F37A Doc2B that we measured by ITC were 0.15 µM and 1.4 µm, respectively (Figure S5), consistent with the involvement of F37 in binding.

### 2.2 Interplay between Interactions of Doc2B with Munc13‐1 and Membranes

Doc2B and Munc13‐1 were shown to co‐translocate to the plasma membrane in response to phorbol esters, which mimic DAG, and to increased intracellular Ca^2+^ [50, 52, 54]. To test whether Munc13‐1 and Doc2B cooperate in membrane binding in vitro, we prepared single cysteine variants of FL Doc2B and Doc2B C_2_AB with the cysteine replacing E266 in the linker between the two C_2_ domains, and labeled them with Alexa‐488 (note that mutation of the native cysteines of Doc2B C_2_AB did not impair folding or its biochemical properties [81]). We then used FRET to monitor binding of 50 nM Alexa‐488‐labeled proteins to liposomes containing DAG and Rhodamine‐phosphatidylethanolamine (PE) (Rho‐liposomes) in the absence and presence of Munc13‐1 C_1_C_2_BMUNC_2_C bearing an R1598E/F1658E mutation (C_1_C_2_BMUNC_2_C^RFEE^). This mutation is in the C_2_C domain and disrupts the liposome clustering activity of C_1_C_2_BMUNC_2_C while preserving the ability of the C_1_‐C_2_B region at the other end of this highly elongated molecule to bind membranes [24], thus preventing interference of liposome clustering with intrinsic binding to a single membrane.

The presence of Munc13‐1 C_1_C_2_BMUNC_2_C^RFEE^ increased the affinity of FL Doc2B but not Doc2B C_2_AB for Rho‐liposomes in the absence of Ca^2+^ (Figure 2A,B). In contrast, C_1_C_2_BMUNC_2_C^RFEE^ did not enhance binding of FL Doc2B to Rho‐liposomes lacking DAG (Figure 2C). These results support the notion that Doc2B and Munc13‐1 cooperate in membrane binding and show that cooperation requires DAG and the Doc2B IDR. Such cooperation most likely arises because binding of the Doc2B MID to the Munc13‐1 MUN domain joins the two proteins together and facilitates simultaneous binding of the Munc13‐1 C_1_ domain to DAG and of the polybasic regions of the Doc2B C_2_ domains to negatively charged lipids. Interestingly, FL Doc2B bound to liposomes with much higher affinity than Doc2B C_2_AB (Figure 2A,B), suggesting that the IDR has an intrinsic membrane‐binding capacity in the absence of Munc13‐1 and may cooperate with the Doc2B C_2_ domains in membrane binding when Munc13‐1 is absent.

**FIGURE 2:**
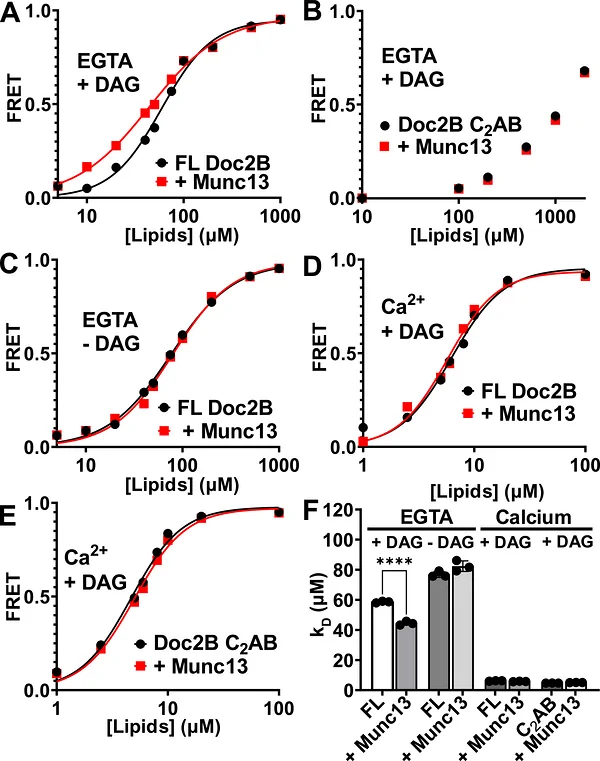
The Doc2B IDR and the IDR‐Munc13‐1 interaction enhance Ca^2+^‐independent binding of Doc2B to membranes. (A–E) FRET was used to monitor binding of 50 nM Alexa‐488‐labeled FL Doc2B (A, C, D), or Doc2B C_2_AB (B, E) to increasing concentrations of Rho‐liposomes containing (A, B, D, E), or lacking (C) 2% DAG in the absence (black symbols) or presence (red symbols) of 55 nM Munc13‐1 C_1_C_2_BMUNC_2_C^RFEE^ (Munc13), and EGTA (A–C), or Ca^2+^ (D, E). Curves show fits with a Hill equation. (*F*) K_D_s obtained from fits of three replicates for each condition. Error bars are SDs. Note that the K_D_ for Doc2B C_2_AB in EGTA is not shown as the affinity was too weak to obtain reliable values. Liposomes were composed of 38% POPC, 18% DOPS, 15% POPE, 20% Cholesterol, 5% Rhodamine‐PE, 2% PIP2, and 2% DAG, which was replaced with POPC for ‘− DAG’ liposomes. Experiments were performed in 100 mM KCl and 1 mM EGTA, or 0.5 mM Ca^2+^. Statistical significance and p values were determined by ordinary one‐way ANOVA with Sidak's multiple comparisons test (****p ≤ 0.0001). Average K_D_s [95% confidence level in brackets] from left to right are 58.6 µM [57.2, 59.9], 44.4 µM [41.3, 47.5], 76.9 µm [72.0, 81.8], 82.3 µM [73.7, 91.0], 6.2 µM [5.7, 6.6], 5.8 µM [5.4, 6.2], 4.7 µM [4.6, 4.8], and 5.1 µM [4.9, 5.3].

In the presence of Ca^2+^, Munc13‐1 C_1_C_2_BMUNC_2_C^RFEE^ did not enhance binding of FL Doc2B to Rho‐liposomes containing DAG (Figure 2D). However, binding was almost saturated at 10 µM lipid concentrations and a large percentage of the liposome surface should be covered by the proteins at these protein‐to‐lipid (P:L) ratios. These observations suggest that saturation binding occurred for isolated FL Doc2B and hence that it was not possible to observe a C_1_C_2_BMUNC_2_C^RFEE^‐induced increase in affinity under these conditions even if the two proteins cooperate in membrane binding. This proposal is supported by the observation that FL Doc2B and Doc2B C_2_AB bound to Rho‐liposomes with similar affinity in the presence of Ca^2+^ (Figure 2D–F), indicating that any enhancement of affinity caused by the IDR was masked because both proteins exhibited saturation binding curves.

Molecular crowding may have also hindered observation of a more dramatic enhancement of the binding of FL Doc2B to DAG‐containing liposomes caused by Munc13‐1 C_1_C_2_BMUNC_2_C in the absence of Ca^2+^ (Figure 2A), but the enhancement was clearly reproducible and significant (Figure 2F). The moderate nature of the increase in affinity may also arise in part if the Doc2B IDR contributes to binding of FL Doc2B to the membrane in the absence of C_1_C_2_BMUNC_2_C but not in its presence, for instance if the MID mediates binding of the IDR to lipids but is sequestered upon binding to the MUN domain. To examine this possibility, we used NMR spectroscopy. Transverse relaxation optimized spectroscopy (TROSY) ^1^H‐^15^N heteronuclear single quantum coherence (HSQC) spectra of ^15^N‐labeled Doc2B IDR exhibited sharp cross‐peaks consistent with its flexible nature, and broadening beyond detection of selected cross‐peaks was observed in the presence of Munc13‐1 MUN domain (Figure 3A). These cross‐peaks correspond to the MID, which becomes immobilized upon binding to MUN whereas cross‐peaks from residues that retain high mobility remain observable. Importantly, similar cross‐peaks of the Doc2B IDR were broadened in the presence of liposomes (Figure 3B), showing that the same region of Doc2B (the MID) binds to the lipids and the MUN domain.

**FIGURE 3:**
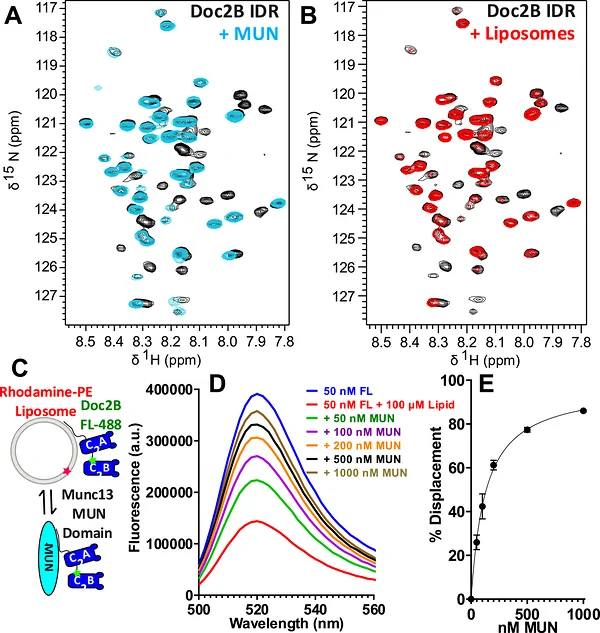
The Doc2B MID binds directly to lipids, which competes with Doc2B MID‐Munc13‐1 MUN domain binding. (A, B) Expansions of ^15^N‐^1^H TROSY‐HSQC spectra of ^15^N‐Doc2B IDR alone (black) or in the presence of Munc13‐1 MUN domain (*A*, cyan) or liposomes (*B*, red). (C) Diagram illustrating the FRET experiments of (D, E). (*D*) Representative fluorescence spectra of 50 nM Alexa‐488‐Doc2B FL alone (blue) or in the presence of Rho‐liposomes (100 µM total lipids) without (red) or with the indicated concentrations of Munc13‐1 MUN domain (color coded). (E) Percentage of displacement of FL Doc2B from liposomes as a function of MUN concentration assessed from the FRET data of (D). Each point is the average of three replicates and the curve shows a fit with a [Inhibitor] vs response model (three parameters). The calculated IC50 [95% confidence level] is 127 nM [96.0, 170.1]. Error bars represent SEMs. Experiments were performed in 100 mM KCl and 1 mM EGTA. Liposomes were composed of 38% POPC, 18% DOPS, 15% POPE, 20% Cholesterol, 5% Rhodamine‐PE, 2% PIP2, and 2% DAG.

This conclusion was further supported by the observation that addition of increasing amounts of MUN domain gradually decreased FRET between 50 nM Alexa‐488 FL Doc2B and Rho‐liposomes (100 µM lipids) (Figure 3C–E), showing that MUN and the liposomes compete for binding to FL Doc2B. Note that MUN does not cooperate with the Doc2B C_2_ domains in membrane binding because it is missing the C_1_‐C_2_B region of Munc13‐1 and the low affinity of the Doc2B C_2_ domains for the Rho‐liposomes is not sufficient for binding to them under these conditions (Figure 2B). The finding that MUN could not displace Doc2B C_2_AB from the liposomes at higher lipid concentrations (1000 µM) (Figure S6A–C) confirmed that the displacement of FL Doc2B from the liposomes is caused by binding of MUN to the IDR. The IC50 observed in the displacement with MUN was 0.13 µM (Figure 3E), which is consistent with the K_D_ between FL Doc2B and MUN measured by ITC (0.15 µM, see above) and shows that MUN competes with high efficiency with the lipids for binding to Doc2B.

We also tested whether pre‐formed FL Doc2B‐MUN complex could be disassembled by liposomes. To this end, we inserted a SNAP‐tag into a flexible loop of the MUN domain (see Supporting Information) and labeled it with Alexa‐546. The labeled protein exhibited a small but reproducible FRET (∼8%–9%) with Alexa488‐FL Doc2B that was lost upon addition of liposomes, but large lipid concentrations (hundreds of micromolar) were required to displace SNAP‐MUN from FL Doc2B (Figure S6D–F). These results show that the Doc2B MID binds much tighter to MUN than to membranes and thus support the notion that the MID remains bound to the Munc13‐1 MUN domain when C_1_C_2_BMUNC_2_C and FL Doc2B co‐translocate to the liposomes (Figure 2A). However, the affinity of the MID for lipids might facilitate transient release of the MID‐MUN interaction to allow MUN to open syntaxin‐1 (see discussion).

### 2.3 Binding of Doc2B to Membrane‐Anchored SNARE Complex

Previous studies showed that the Ca^2+^‐stimulated binding of Syt1 C_2_AB to the SNARE complex in solution is misleading, as Ca^2+^ actually decreases the affinity of Syt1 C_2_AB for the SNARE complex on membranes [63]. It is unclear whether this behavior is shared by other proteins containing tandem C_2_ domains. To test whether Doc2B C_2_AB binds to membrane‐anchored SNARE complex in the absence of Ca^2+^, we monitored FRET from Alexa488‐Doc2B C_2_AB to SNARE complex anchored on liposomes and labeled at residue 76 of SNAP‐25 with tetramethylrhodamine (TMR) (Figure 4A) as we used for Syt1 studies [63]. The SNARE complex was reconstituted into the liposomes at a low P:L ratio (1:2000) to minimize the possibility of observing FRET due to random encounters between Alexa488‐Doc2B C_2_AB and TMR‐SNARE complex on the liposome surface. Clear binding was observed, with an apparent K_D_ of 0.91 µM (Figure 4B,C). Binding appears to be relatively weak, since Doc2B C_2_AB also binds to protein‐free liposomes at comparable lipid concentrations (Figure 2B), but binding of FL Doc2B to the SNARE complex in neurons is most likely enhanced by co‐localization at the plasma membrane.

**FIGURE 4:**
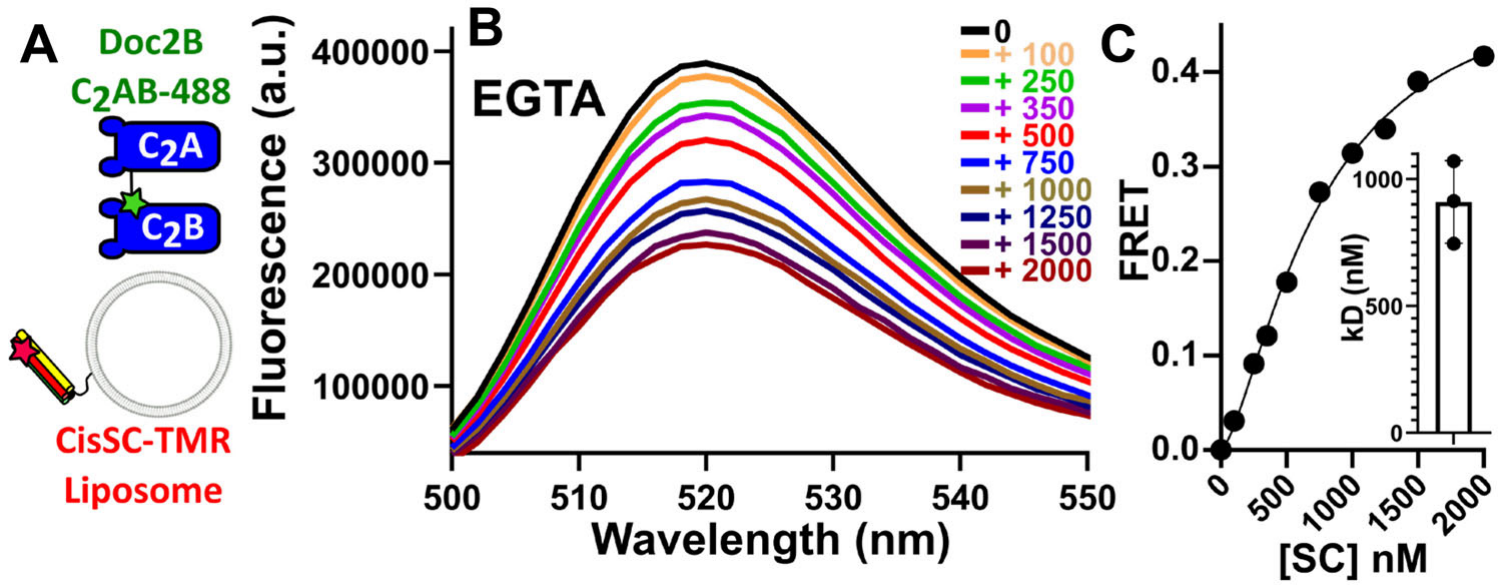
Doc2B C_2_AB binding to liposome‐anchored SNARE complex. (A) Setup of the FRET experiments of (B). (B) Representative fluorescence spectra of 50 nM Alexa‐488 Doc2B C_2_AB in the presence of 1 mM EGTA and the indicated concentrations (nM) of TMR‐labeled SNARE complex (CisSC‐TMR) anchored on liposomes (1:2000 P:L ratio). (C) Plot of FRET as a function of SNARE complex concentration (SC) from the data of (B). The curve is a fit to a Hill equation. The inset shows a bar diagram representing the average of the K_D_s measured in three replicates (error bar refers to SD). Average K_D_ [95% confidence level] = 910 nM [506, 1313]. Experiments were performed in 100 mM KCl and 1 mM EGTA. Liposomes were composed of 38% POPC, 18% DOPS, 20% POPE, 20% Cholesterol, 2% PIP2, and 2% DAG.

In initial experiments performed in the presence of Ca^2+^, no apparent FRET was observed between Alexa488‐Doc2B C_2_AB and TMR‐SNARE complex liposomes even at 0.5 µM SNARE complex‐liposomes (Figure S7A,B), which corresponds to a 1000 µM lipid concentration at which Alexa488‐Doc2B C_2_AB should be quantitatively bound to the liposomes (Figure 2E). To investigate the Ca^2+^‐dependence of the loss of FRET, we used Indo‐1 as an internal indicator of Ca^2+^ concentration (Figure S7C,D) with an EGTA‐based Ca^2+^ buffering system and observed a bimodal curve with negative FRET at ca. 33 µM Ca^2+^ (Figure S7E, gray circles). Parallel experiments without the TMR probe on the SNARE complex revealed a Ca^2+^‐dependence of the Alexa488‐Doc2B C_2_AB fluorescence (Figure S7F) that suggests a change of the probe environment due Ca^2+^‐induced structural rearrangements. FRET remained after correcting for this phenomenon (Figure S7E, black circles). We note that the Alexa488‐Doc2B C_2_AB fluorescence was not affected by unlabeled SNARE‐complex liposomes in the absence of Ca^2+^ (Figure S7F), showing that the observed FRET (Figure 4B,C) is not artifactual. Overall, these results show that Ca^2+^‐free Doc2B C_2_AB binds to membrane‐anchored SNARE complex and suggest that Ca^2+^ induces rearrangements that will need to be studied in the future with probes in distinct locations and/or alternative approaches.

### 2.4 Doc2B‐SNARE Complex Binding Mode

Determining the physiologically relevant binding mode between the Syt1 C_2_B domain and the SNARE complex involving the primary interface [25] took over two decades because of the promiscuity of these proteins and the fact that the C_2_B polybasic region that binds to PIP_2_ on the membrane [63, 82] dominates binding to the SNARE complex in solution [83, 84]. The participation of the Syt1 C_2_A domain in binding is still unclear [65]. To gain insights into how Doc2B binds to the SNARE complex, we used NMR spectroscopy and a Doc2B C_2_AB fragment (C_2_AB^KKQQ^) bearing a K316Q,K319Q mutation in the C_2_B polybasic region homologous to that of Syt1 C_2_B.

^1^H‐^15^N TROSY‐HSQC spectra of ^15^N‐labeled C_2_AB^KKQQ^ exhibited progressive broadening of many cross‐peaks with increasing concentrations of soluble SNARE complex (Figure S8), indicative of binding. To test which domain mediates binding, we acquired ^1^H‐^15^N TROSY‐HSQC spectra of the isolated Doc2B C_2_A and C_2_B^KKQQ^ domains. The SNARE complex caused shifts of multiple cross‐peaks of C_2_A but no overt shifts for C_2_B^KKQQ^ (Figures S9A and S10). We assigned most of the ^1^H‐^15^N TROSY‐HSQC cross‐peaks of C_2_A using triple resonance experiments (Figure S9B), and mapping the cross‐peaks that exhibited substantial SNARE‐induced broadening showed that binding was mediated by the concave side of the C_2_A β‐sandwich (Figure S9C). Analysis of the charge distribution of C_2_A showed that this concave side is also polybasic and resembles those of Syt C_2_B domains (Figure S9D). These observations suggest that this region of C_2_A should also bind to PIP_2_ in membranes that mimic the plasma membrane and hence that the observed binding to the soluble SNARE complex is dominated by irrelevant interactions. Indeed, a K176Q,K179Q mutation in the polybasic region of the C_2_A domain (C_2_A^KKQQ^) severely impaired Ca^2+^‐independent binding of Alexa‐488 C_2_AB Doc2B to Rho‐liposomes, similar to the impairment caused by the K316Q,K319Q mutation in C_2_B (Figure S11). These results suggest that the polybasic regions of both C_2_ domains cooperate in binding of Doc2B C_2_AB to membranes.

In solution, ^1^H‐^15^N TROSY‐HSQC spectra showed that the K176Q, K179Q mutation practically abolished binding of C_2_A to the SNARE complex (Figure S12). Interestingly, the SNARE complex caused shifts in selective cross‐peaks of ^1^H‐^15^N TROSY‐HSQC spectra of Doc2B C_2_AB bearing the K176Q, K179Q mutation in C_2_A and the K316Q, K319Q mutation in C_2_B (C_2_AB^KKQQ, KKQQ^) (Figure 5A and Figure S13) and the shifted cross‐peaks were from C_2_B residues. We assigned most of the cross‐peaks of C_2_B^KKQQ^ using triple resonance experiments (Figure S14), which showed that the cross‐peaks that shift upon binding of C_2_AB^KKQQ, KKQQ^ to the SNARE complex are from the two helices at the tip of the C_2_B β‐sandwich opposite to its Ca^2+^‐binding sites (Figure 5B). Note that we did not observe cross‐peak shifts when we tested binding of the SNARE complex to C_2_B^KKQQ^ (Figure S10) but the overall positive charge of C_2_A^KKQQ^ likely increased the affinity of C_2_AB^KKQQ, KKQQ^ for the SNARE complex (compared to C_2_B^KKQQ^), as the overall electrostatic potential is important for binding to the negatively charged SNARE complex [65].

**FIGURE 5:**
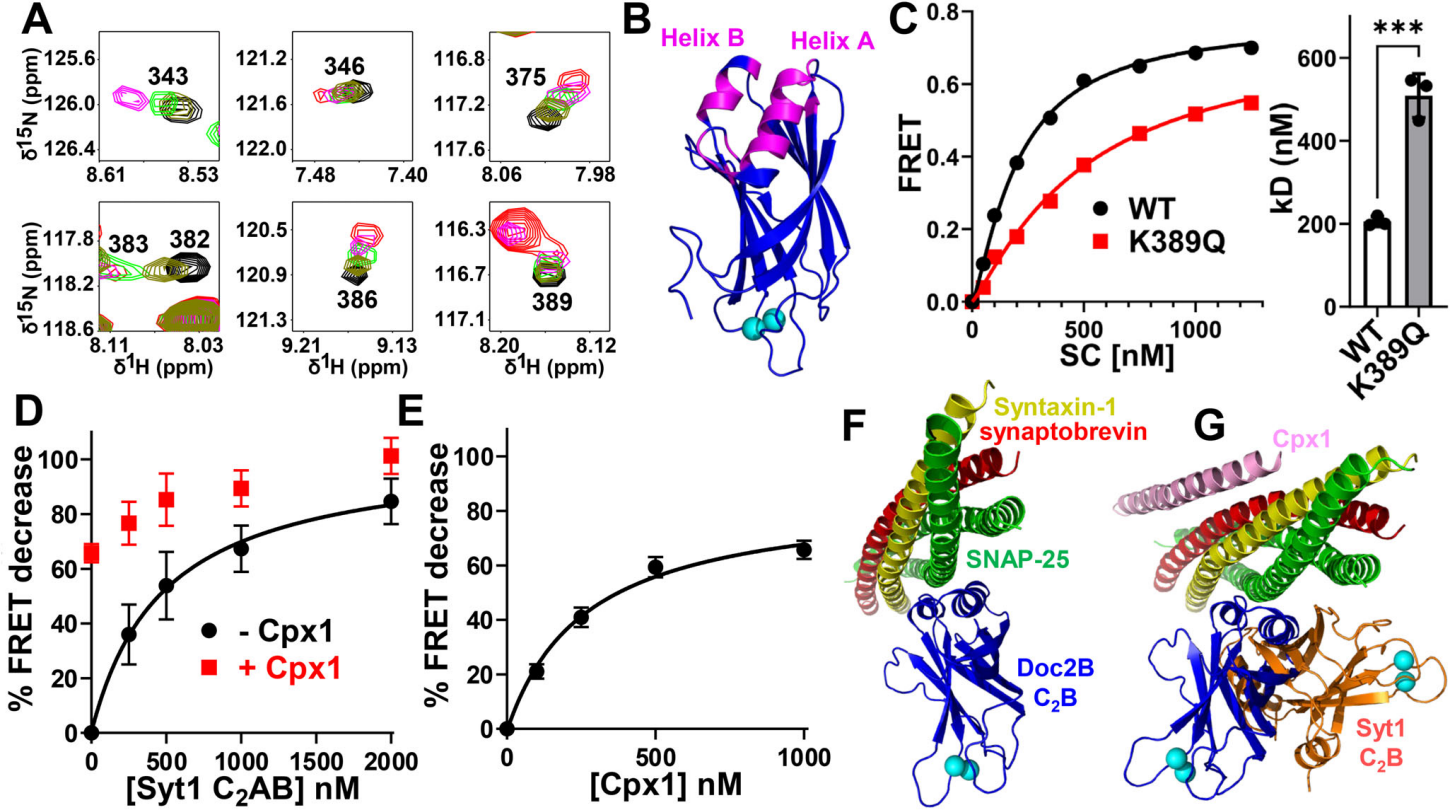
Doc2B binds to the SNARE complex through two helices at one tip of the β‐sandwich. (A) Expansions of ^15^N‐^1^H TROSY‐HSQC of 70 µM ^15^N‐Doc2B C_2_AB^KKQQ, KKQQ^ alone (black) or after adding 20, 50, 80, or 120 µM soluble SNARE complex (bronze, green, magenta and red, respectively). Contour levels were adjusted manually to visualize shifts. (B) Ribbon diagram of the crystal structure of the Doc2B C_2_B domain [60] (PDB accession code 4LDC) with residues exhibiting substantial SNARE complex‐induced shifts in the ^1^H‐^15^N TROSY‐HSQC spectra of (A) and Figure S13 colored in magenta (Δδ^1^H > 0.02 ppm and/or Δδ^15^N > 0.2 ppm). Ca^2+^ ions are shown as cyan spheres. (*C*) Plot of the FRET between 50 nM Alexa‐488 WT (black) or K389Q (red) Doc2B C_2_AB and liposome‐anchored TMR‐SNARE complex (1:2000 P:L ratio) as a function of SNARE complex concentration (SC) in 50 mm KCl and 1 mM EGTA. The curve shows a fit to a Hill equation. The bar diagram on the right shows the average K_D_s obtained in three replicates [95% confidence level]—WT: 206 nM [175, 236]; K389Q: 509 nM [378, 640]. Statistical significance and p value were determined by unpaired two‐tailed t‐test (***p ≤ 0.001). (*D, E*) Percentage of FRET decrease between 50 nM Alexa‐488‐Doc2B C_2_AB and 900 nM TMR‐SNARE complex anchored on liposomes (1:2000 P:L ratio) as a function of Syt1 C_2_AB concentration in the absence (black symbols) or presence (red symbols) of 1 µM Cpx1 (D), or as a function of Cpx1 concentration (E), in 100 mM KCl and 1 mM EGTA. Each point represents the mean of three replicates and error bars represent standard deviations. Curves show fits of the data with an [Inhibitor] vs response model (three parameters). Calculated IC50s [95% confidence level]: Cpx1, 266 nM [192, 376]; Syt1 without Cpx1, 480 nM [246, 1004] (no reliable K_D_ can be obtained with Cpx1). Liposome composition for (C–E) was the same as in Figure 4. (F) Ribbon diagram of the SNARE complex bound to Doc2B C_2_B based on the crystal structure of the rabphilin 3A C_2_B‐SNAP‐25 complex [87] (PDB accession code 5LOB). (G) Superposition of the model of (F) with the crystal structures of the SNARE complex bound to Cpx1(26–83) [26] or Syt1 C_2_B [25] (PDB accession codes 1KIL and 5KJ7, respectively).

We did not attempt to perform the reciprocal experiments with isotopically labeled SNAREs to map the binding site of Doc2B on the SNARE complex because its ^1^H‐^15^N TROSY‐HSQC spectra did not exhibit substantial shifts upon binding to Syt1 C_2_AB [84, 85]. However, it is noteworthy that NMR spectroscopy and X‐ray crystallography showed that the C_2_B domain of rabphilin 3A, another tandem C_2_ domain protein involved in membrane traffic, binds SNAP‐25 through the same two helices involved in binding of the Doc2B C_2_B domain to the SNARE complex [86, 87]. Moreover, the sequences of the two helices of rabphilin 3A are highly conserved in Doc2B, including key residues that contact SNAP‐25 in the SNAP‐25‐rabphilin 3A C_2_B crystal structure [87] such as those corresponding to F385 and K389 of Doc2B (Figure S15). These results suggest that Doc2B C_2_B binds to SNAP‐25 in the SNARE complex through the mode observed in that crystal structure. To verify that the binding mode observed in solution also occurs on membranes, we used our FRET assays that monitor binding of Alexa488‐Doc2B C_2_AB to liposome‐anchored TMR‐SNARE complex, but with low salt concentration (50 mM) to enhance affinity and facilitate quantitative analysis of mutational effects. We found that a K389Q mutation in the C_2_B helix markedly impaired the binding of Doc2B C_2_AB to liposome‐anchored SNARE complex (Figure 5C), confirming the participation of this helix in SNARE binding.

### 2.5 Competition of Syt1 and Complexin with Doc2B for SNARE Binding

Primed SNARE complexes that are ready for synchronous neurotransmitter release are bound to Syt1 and to complexin [25, 27]. Can these interactions occur simultaneously with binding of Doc2B to the SNARE complex? To address this question, we set up competition assays with 50 nM Alexa‐488‐Doc2B C_2_AB and 0.9 µM TMR‐SNARE complex anchored on liposomes such that there is substantial FRET (Figure 4C). Gradual addition of Syt1 C_2_AB revealed progressive loss of FRET, indicating displacement of Alexa‐488‐Doc2B C_2_AB from the SNARE complex with an EC50 of 0.48 µM (Figure 5D). Complexin‐1 (Cpx1), which binds with 10 nm affinity to the SNARE complex [88], also decreased the FRET but not completely even at 1 µM concentration, which should saturate the SNARE complex (Figure 5E). Hence, Cpx1 appears to distort the Doc2B C_2_AB‐SNARE complex without dissociating it. Interestingly, competition with Syt1 C_2_AB in the presence of 1 µM Cpx1 led more efficiently to full displacement of Doc2B C_2_AB than in the absence of Cpx1 (Figure 5D).

These findings can be rationalized with a model of the Doc2B C_2_B‐SNARE complex based on the rabphilin 3A‐SNAP‐25 crystal structure (Figure 5F). Comparison with the crystal structure of the SNARE complex bound to Syt1 C_2_B [25] predicts that Doc2B and Syt1 cannot bind simultaneously to the SNARE complex because, while they use different surfaces to bind to SNAP‐25, the same region of SNAP‐25 binds to the two proteins (Figure 5G). These observations explain the displacement of Doc2B C_2_AB from the SNARE complex by Syt1 C_2_AB. We note that Syt1 C_2_B was found to bind to a different site of the SNARE complex via the two helices in a crystal structure [89], but this interaction was not observed on membranes or in solution even with highly sensitive methods [63, 90] and hence cannot explain the competition data of Figure 5D.

Comparison of the Doc2B C_2_B‐SNARE complex model with the crystal structure of the SNARE complex bound to a Cpx1 fragment [26] (Figure 5G) shows that the binding sites on the SNARE complex are distant. A potential explanation for the disruption of Doc2B C_2_AB‐SNARE complex binding by Cpx1 (Figure 5E) is offered by modeling how the Doc2B C_2_B domain interacts with membrane‐anchored SNARE complex while the C_2_B polybasic region binds to the membrane, which would likely cause steric clashes of Cpx1 with the membrane (Figure S16 and Supporting Information). However, other explanations are possible. Regardless of the explanation, the notion that Cpx1 does not compete with Doc2B C_2_AB for binding to the soluble SNARE complex was supported by the observation that the SNARE complex causes comparable cross‐peak broadening in ^1^H‐^15^N TROSY‐HSQC spectra of C_2_AB^KKQQ, KKQQ^ in the absence and presence of Cpx1, showing that C_2_AB^KKQQ, KKQQ^ is not displaced from the soluble SNARE complex by Cpx1 (Figure S17).

### 2.6 Functional Interplay between Doc2B Interactions

To explore the functional interplay among the various interactions of Doc2B, we studied the effects of WT and mutant Doc2B in our well‐established reconstitution assays, which analyze fusion between synaptobrevin‐containing liposomes (V‐liposomes) and liposomes containing syntaxin‐1 and SNAP‐25 (T‐liposomes) in the presence of Munc18‐1, Munc13‐1 C_1_C_2_BMUNC_2_C, NSF, and αSNAP [23]. In these assays, fusion is dramatically stimulated by Ca^2+^ because it activates the Munc13‐1 C_2_B domain, strongly enhancing catalysis of SNARE complex assembly by C_1_C_2_BMUNC_2_C [14] and mimicking in part the enhancement of neurotransmitter release caused by Ca^2+^ accumulation during repetitive stimulation [38, 91].

To facilitate analysis of roles performed by Doc2B before Ca^2+^ binding, we anchored SNAP‐25 on the T‐liposomes by dodecylation [92], which allows observation of Ca^2+^‐independent fusion but also renders Ca^2+^‐dependent fusion very efficient [7] and can mask moderate impairments of the efficiency of Ca^2+^‐dependent fusion caused by mutations. Thus, we adjusted the concentration of C_1_C_2_BMUNC_2_C to allow observation of effects of Doc2B mutations on both components. Under these conditions, the natural variability of the data hindered observation of statistically significant effects for some of the mutations (Figure 6 and Figure S18), but important insights can still be obtained from the tendencies observed and the overall data. Interpretation of the data is also hindered by the fact that these assays do not distinguish among the steps leading to liposome fusion, which include initial tethering between V‐ and T‐liposomes, orchestration of SNARE complex assembly and membrane fusion among others. Moreover, some of the proteins play both inhibitory and stimulatory roles that can be altered by Ca^2+^. Because of this complexity, there are likely multiple ways to explain the results of our fusion assays, but we offer plausible interpretations that are based on the known biochemical properties of the components. In this context, it is important to consider that the Doc2B MID inhibits syntaxin‐1 opening by C_1_C_2_BMUNC_2_C [57] but helps to co‐translocate Doc2B with C_1_C_2_BMUNC_2_C to liposomes in the absence of Ca^2+^ (Figure 2A). Although Ca^2+^ can induce co‐translocation of Doc2B and Munc13‐1 in cells [50], cooperation between Ca^2+^‐bound Doc2B and C_1_C_2_BMUNC_2_C in liposome binding cannot be observed at the concentrations used for our experiments (Figure 2D) due to their high membrane affinity upon Ca^2+^ binding. Note also that, because binding of the MID to Munc13‐1 is tighter than to liposomes (Figure 3E, Figures S5A, and S6F), the MID is expected to remain mostly bound to Munc13‐1 C_1_C_2_BMUNC_2_C when both proteins bind to the liposomes. However, the proximity to the lipids may facilitate MID‐membrane interactions that can occur transiently, leaving the Munc13‐1 NF pocket temporarily vacant and thus allowing syntaxin‐1 binding and opening.

**FIGURE 6:**
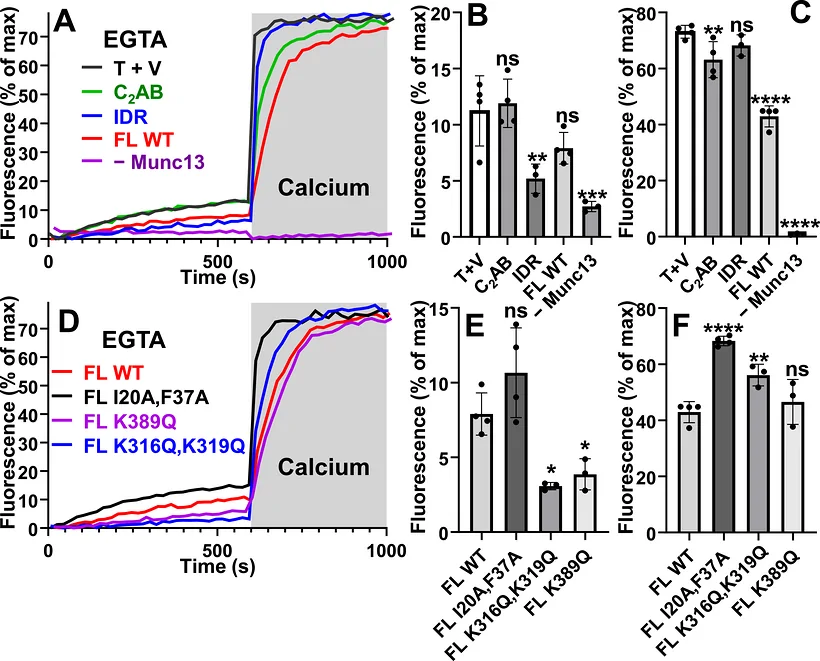
Modulation of SNARE and Munc13‐1‐dependent liposome fusion by Doc2B. (A, D) Content mixing between V‐ and T‐liposomes was measured from the development of FRET between Cy5‐Streptavidin trapped in the V‐liposomes and Phycoerythrin‐biotin‐XX conjugate trapped in the T‐liposomes. The assays were performed in the presence of Munc18‐1, NSF, αSNAP, and Munc13‐1 C_1_C_2_BMUNC_2_C in the absence (T+V) or presence of FL Doc2B or the indicated Doc2B fragments. “‐Munc13” refers to a negative control analogous to T + V but with Munc13 omitted. Experiments were started in the presence of 100 µM EGTA and 5 µM streptavidin, and Ca^2+^ (600 µM) was added after 600 s. (B, C, E, F) Quantification of the content mixing observed in EGTA after 500 s (B, E) and in Ca^2+^ after 11 min (C, F). Error bars are standard deviations. Statistical significance and p values with respect to T+V (B, C) or WT FL Doc2B (E, F) were determined by ordinary one‐way ANOVA with Holm‐Sidak's multiple comparisons test (ns = not significant; *p ≤ 0.05; **p ≤ 0.01; ***p ≤ 0.001; ****p ≤ 0.0001).

In the absence of Ca^2+^, liposome fusion was not affected by Doc2B C_2_AB but was impaired by the IDR, as expected because the MID blocks the Munc13‐1 MUN domain (Figure 6A,B and Figure S18A,B). FL Doc2B appeared to inhibit fusion but less efficiently than the IDR, which may arise because the inhibition caused by the MID may be compensated partially by a positive role of FL Doc2B in helping to localize Munc13‐1 C_1_C_2_BMUNC_2_C to the T‐liposomes. FL Doc2B bearing an I20A, F37A mutation designed to disrupt MID‐MUN binding [57] (Figure 1E and Figure S5) did not appear to inhibit fusion (compare FL I20A, F37A in Figure 6E with T+V in Figure 6B), as expected. However, the inhibition of content mixing caused by FL Doc2B was enhanced by the K316Q, K319Q mutation and the K389Q mutation in C_2_B (Figure 6D,E). These results likely arose because of impaired binding of FL Doc2B to the T‐liposomes, as the former mutation disrupts binding of the C_2_B domain to lipids and the latter is expected to impair binding of C_2_B to SNAP‐25, and both interactions likely cooperate in FL Doc2B binding to T‐liposomes.

In the presence of Ca^2+^, Doc2B C_2_AB appeared to inhibit fusion slightly (Figure 6A,C and Figure S18A,C), perhaps because it competes with Munc13‐1 C_1_C_2_BMUNC_2_C for binding to PIP_2_ in the T‐liposomes, which helps activate the Munc13‐1 C_2_B domain [23, 38]. Surprisingly, we did not observe inhibition by the IDR, but there was a substantial inhibition by FL Doc2B. These results contrast with the finding that both the IDR and FL Doc2B almost abolished fusion supported by Munc13‐1 C_1_C_2_BMUN [57], which most likely arises because this fragment used in [57] is much less active than C_1_C_2_BMUNC_2_C [23]. Our results can be rationalized by a model whereby C_1_C_2_BMUNC_2_C is very active in the presence of Ca^2+^ and soluble IDR does not fully saturate the MUN domain such that any inhibition of C_1_C_2_BMUNC_2_C by the IDR is masked by the high efficiency of the C_1_C_2_BMUNC_2_C that remains free. In this model, FL Doc2B inhibits fusion in part because it competes with C_1_C_2_BMUNC_2_C for PIP_2_ binding, as C_2_AB does, and in part because FL Doc2B binds quantitatively to the membrane through its tandem C_2_ domains, increasing the local IDR concentration and saturating C_1_C_2_BMUNC_2_C more fully. Note that, in contrast to the Ca^2+^‐free conditions, C_1_C_2_BMUNC_2_C is expected to bind quantitatively to the liposomes in the presence of Ca^2+^ [5, 23] and hence there is no beneficial role for Doc2B in attracting C_1_C_2_BMUNC_2_C to the T‐liposomes. As expected, the I20A, F37A mutation designed to disrupt MID‐MUN binding abrogated the inhibition by FL Doc2B (Figure 6D,F and Figure S18D,F), supporting the notion that the inhibition arises because the MID blocks the MUN domain. The K316Q, K319Q mutation decreased the inhibitory activity of FL Doc2B (Figure 6D,F, Figure S18D,F), since it disrupts PIP_2_ binding and decreases the affinity for the T‐liposomes. In contrast, the K389Q mutation did not alter the activity of FL Doc2B (Figure 6D,F and Figure S18D,F), perhaps because K389 does not contribute to SNARE binding when Ca^2+^ rearranges the complex and hence to the affinity for the T‐liposomes in the presence of Ca^2+^.

As mentioned above, there are likely alternative potential interpretations of these results but, overall, they clearly support the notion that Doc2B function depends on interactions with Munc13‐1, the SNAREs, the lipids and Ca^2+^.

## 3 Discussion

Ample evidence showed that Doc2s play diverse functions in neurotransmitter release and presynaptic plasticity, and that mutations in these proteins cause diverse neurologic and psychiatric disorders. It was also clear that the functions of Doc2s depend on their interactions with Munc13s, SNAREs, membranes and Ca^2+^. However, the underlying molecular mechanisms remained enigmatic, in large part because there was little information on the interplay among these interactions, no structures of complexes of Doc2s with Munc13‐1 or the SNAREs were available, and in fact it was unclear whether Doc2s bind to SNAREs in a membrane environment. Our cryo‐EM structure now reveals how the Doc2B MID binds to Munc13‐1 and explains why Doc2B inhibits SNARE complex assembly. We also show that Doc2B and Munc13‐1 bind synergistically to membranes in the absence of Ca^2+^ and identify a novel interaction of the Doc2B MID with lipids that competes with MUN binding and cooperates with the C_2_ domains in Ca^2+^‐independent membrane binding. Moreover, our data show that Doc2B binds to the SNARE complex via two helices of the C_2_B domain in the absence of Ca^2+^, competing with Syt1 and Cpx1 for binding to membrane‐anchored SNARE complex. These results are critical to understand Doc2 function and, together with previous studies, lead us to propose a speculative working model that includes the following premises (Figure 7): 1. Doc2B enhances translocation of Munc13‐1 to the plasma membrane (Figure 7A,B), which is stimulated by Ca^2+^ accumulation during presynaptic plasticity. 2. Binding of the Doc2B C_2_ domains to the membrane facilitates binding of C_2_B to SNAP‐25 and binding of the MID to lipids that may catalyze transient release of its interaction with the MUN domain; Munc13‐1 can then mediate opening of syntaxin‐1 to form the template complex (Figure 7C), leading to SNARE complex assembly with Doc2B bound (Figure 7D). Doc2B binding to the SNARE complex is proposed to be part of a primed state that underlies Doc2B‐dependent forms of release by as yet unclear mechanisms. In this model, Doc2B acts as a Ca^2+^ sensor in the last steps leading to release and also by mediating Ca^2+^‐dependent co‐translocation to the plasma membrane with Munc13‐1.

**FIGURE 7:**
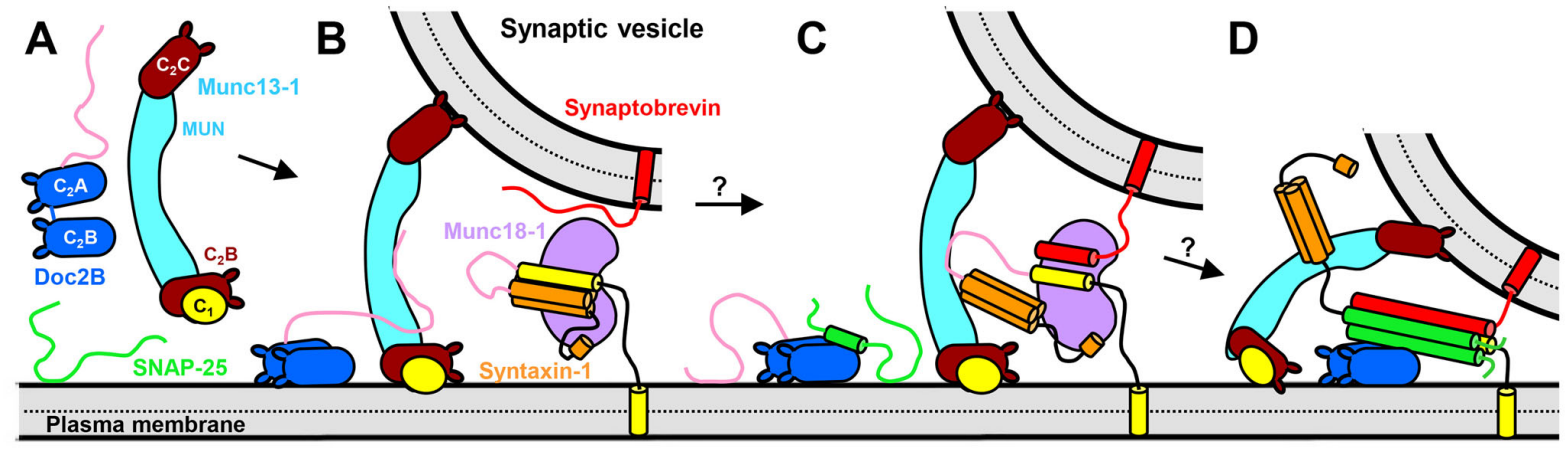
Speculative working model of how Doc2B interactions control distinct steps leading to neurotransmitter release. In this model, Munc13‐1 and Doc2B are soluble proteins (A) but binding of the Doc2B MID to the Munc13‐1 MUN domain facilitates co‐translocation of both proteins to the plasma membrane by promoting cooperation of interactions of the plasma membrane with the Doc2B C_2_ domains and the Munc13‐1 C_1_ and C_2_B domains (B). Such co‐translocation should be even more efficient in the presence of Ca^2+^, which enhances binding of both proteins to the membrane. Plasma membrane localization stimulates interactions of the Munc13‐1 C_2_C domain with the vesicle, such that Munc13‐1 bridges the two membranes (B). Transient binding of the Doc2B MID to the plasma membrane may relieve the inhibition of MUN and allow binding of MUN to the syntaxin‐1 linker to stimulate syntaxin‐1 opening and formation of the syntaxin‐1‐Munc18‐1‐synaptobrevin template complex (C). Assembly of the primed trans‐SNARE complex (D) may be catalyzed in part by binding of Doc2B to SNAP‐25 (C, D). The interaction of Doc2B with the SNARE complex inhibits membrane fusion and hence neurotransmitter release (D), but this assembly is primed to participate in spontaneous and other forms of release by as yet unknown mechanisms. Please note that the proposed order of events remains to be well established and that the transitions from panels B to C and C to D are particularly speculative, hence the question marks (see discussion for further details). The color coding for syntaxin‐1 and Munc13‐1 is as described in Figure 1A. Other protein colors are indicated in panels (A, B) of this Figure.

### 3.1 Doc2B MID Binding to Munc13‐1 and to Membranes

Pioneering studies established that binding of the Doc2B MID to the Munc13‐1 MUN domain inhibits the ability of Munc13‐1 to mediate SNARE complex assembly, liposome fusion, and neurotransmitter release [57]. However, the structural basis for these findings was unknown and it was unclear why, despite this inhibitory activity, Doc2s play active roles in spontaneous (and perhaps asynchronous) release, synaptic augmentation, long‐term potentiation and superpriming in neurons, as well as priming in chromaffin cells [46, 47, 48, 50, 51, 52]. The cryo‐EM structure presented here now shows how the MID binds to pockets and grooves along the MUN domain, adopting an irregular elongated conformation with two helical turns. This binding mode occludes the NF motif of the Munc13‐1 MUN domain, which is essential for the activity of Munc13‐1 in opening syntaxin‐1 and for neurotransmitter release [22]. Hence, our structure explains why the Doc2 MID inhibits SNARE complex assembly [57]. This inhibitory mechanism is further supported by AlphaFold3 predictions suggesting that the region around the NF motif involved in Doc2B binding in our cryo‐EM structure also binds to the syntaxin‐1 linker to open its conformation [80]. And yet, MID does not merely play an inhibitory role, as it is known to be critical for the active functions of Doc2B in neurotransmitter release, presynaptic plasticity and priming in chromaffin cells [50, 52, 57].

A potential answer to this apparent paradox was provided by the finding that Doc2B and Munc13‐1 co‐translocate to the plasma membrane [50, 54], which should enhance the ability of Munc13‐1 to perform its essential priming function. But then, how is the inhibition of MUN by the MID relieved? Our discovery that the MID binds to lipids and that this interaction competes with MID‐MUN binding (Figure 3) provides a natural mechanism to disinhibit the MUN domain. In principle, a majority of the MID is expected to remain bound to Munc13‐1 when both proteins bind to a membrane, as the Doc2B IDR is necessary for the synergy between Munc13‐1 and FL Doc2B in liposome binding (Figure 2A,B) and the MID binds to MUN much tighter than to lipids (Figure 3E and Figures S5A and S6F). Note also that the IC50 for displacement of liposomes from FL Doc2B by the MUN domain (Figure 3E) is similar to the K_D_ of the MUN‐MID interaction (Figure S5A and [57]). However, even if a majority of the MID remains bound to MUN upon co‐translocation of Doc2B and Munc13‐1 to a membrane, MID‐lipid interactions may be facilitated by proximity to the membrane and may transiently leave the Munc13‐1 NF motif region free to participate in syntaxin‐1 opening (Figure 7B,C). Hence, these ideas provide an attractive mechanism for how Doc2s can play positive roles in exocytosis despite having a sequence, the MID, that in principle inhibits Munc13‐1, much like other proteins play inhibitory and active roles in release [2].

### 3.2 Doc2B‐SNARE Complex Interactions

Doc2s were shown to bind to the SNARE complex through the polybasic region of the C_2_B domain and binding was enhanced by Ca^2+^ [46, 52], mirroring the behavior of Syt1 [2, 58, 84]. However, it recently became clear that the results with Syt1 arose from the use of soluble proteins and that physiologically relevant binding of Syt1 to membrane‐anchored SNARE complex involves the primary interface [25] while the C_2_B polybasic region binds to PIP_2_ on the plasma membrane [63]. Ca^2+^ actually weakens binding of Syt1 to membrane‐anchored SNARE complex [63], likely causing a reorientation of the Syt1 C_2_B domain that pulls the SNARE complex to trigger fast neurotransmitter release [65, 66]. This mechanism is not expected to be shared generally by most Syt isoforms or other tandem C_2_ domain proteins such as Doc2s because the primary interface is not conserved in them. Hence, it was unclear whether Doc2s actually bind to membrane‐anchored SNARE complex. Our NMR and FRET data now show that Doc2B binds to the SNARE complex via two helices at one tip of the β‐sandwich (Figure 5), which exhibit clear sequence similarity to those of the rabphilin 3A C_2_B domain (Figure S15). These findings suggest that Doc2B binds to SNAP‐25 alone or within the SNARE complex through the same binding mode defined for rabphilin 3A by X‐ray crystallography [87] (Figure 5F). This proposal is supported by the observation that Syt1 C_2_AB competes with Doc2B C_2_AB for SNARE complex binding (Figure 5D), as the Syt1 C_2_B domain binds to the same region of SNAP‐25 albeit through a different surface [25] (Figure 5G). The affinity of the interaction between Doc2B C_2_AB and the SNARE complex is rather weak, even when the SNARE complex is anchored on a membrane (Figure 4C), raising the question as to whether such a weak interaction can be physiologically relevant. However, Syt1 C_2_AB binding to the membrane‐anchored SNARE complex, which is crucial for evoked release [25, 66], is also rather weak [63] and has comparable affinity to that of Doc2B C_2_AB (Figure 4C). Moreover, MID‐lipid interactions are expected to increase the local concentration of Doc2B on the membrane and thus facilitate SNARE binding (Figure 7C).

Doc2B localized to the plasma membrane is likely to bind to SNAP‐25 via the two C_2_B helices and induce formation of helical structure in SNAP‐25 [which is unfolded in isolation [93]], facilitating presentation of SNAP‐25 to the nearby syntaxin‐1‐Munc18‐1‐synaptobrevin template complex (Figure 7C) for SNARE complex assembly (Figure 7D). This model is supported by the finding that Doc2B C_2_AB promotes SNARE complex assembly [48] and by results in chromaffin cells showing that Doc2B plays a role in priming [52]. This role depends on SNARE binding and acts downstream of a distinct priming role of Doc2B that may arise because it helps bring Munc13‐2 to the plasma membrane [52]. Similarly, Syt1 also promotes SNARE complex assembly [14] and plays a role in synaptic vesicle priming [94]. These findings suggest that Doc2B can activate the release machinery both by accelerating SNARE assembly and by promoting translocation of Munc13‐1 to the plasma membrane, which is enhanced by DAG and Ca^2+^ during presynaptic plasticity. These observations emphasize the complexity of Ca^2+^‐regulated exocytosis and can explain the variety of phenotypes observed in the absence of Doc2s. For instance, the observation of a role for Doc2s in vesicle priming in chromaffin cells [52] but not at neuronal synapses [46] may arise because in synapses there is more redundancy for the roles of Doc2s and Syt1 in priming. Note also that the role of Doc2s in augmentation was proposed to arise because they promote translocation of Munc13‐1 to the plasma membrane upon accumulation of Ca^2+^ during repetitive stimulation, leading to superpriming of a population of vesicles to a state with a higher Ca^2+^ sensitivity than normal primed vesicles [50]. However, it cannot be ruled out that Doc2B‐mediated SNARE complex assembly also plays a role in augmentation.

The fact that Ca^2+^ alters the fluorescence of Alexa488‐Doc2B C_2_AB in our FRET studies of its interactions with liposome‐anchored SNARE complex (Figure S7F) hindered interpretation of the FRET data. After correcting for this phenomenon, the dependence of the FRET on Ca^2+^ concentration still exhibited a bimodal shape (Figure S7E) that may arise from enhanced binding of Alexa488‐Doc2B C_2_AB to the SNARE‐complex‐liposomes at submicromolar Ca^2+^ concentrations and Ca^2+^‐induced rearrangements that may occur at submicromolar and low micromolar Ca^2+^. This bimodal behavior may arise from the fact that the Doc2B C_2_B domain has a much higher Ca^2+^ affinity than C_2_A [60] but, clearly, further research will be necessary to elucidate the effects of Ca^2+^ on Doc2B‐SNARE complex interactions. It is worth noting that Ca^2+^‐dependent insertion of the Ca^2+^‐binding loops of the Syt1 C_2_ domains into membranes was widely believed to facilitate membrane fusion by perturbing the bilayers [95, 96, 97] and/or inducing membrane curvature [98, 99], and that proteins with tandem C_2_ domains such as Doc2s were believed to stimulate membrane fusion by analogous mechanisms. However, molecular dynamics (MD) simulations showed that placing the Syt1 C_2_ domains near the site of fusion strongly hinders [100] the actions of the SNARE complex that lead to membrane fusion [12]. These observations explained why the C_2_B domain Ca^2+^‐binding loops point away from the fusion site when Syt1 binds to the SNARE complex through the primary interface and led in part to the notion that Syt1 acts remotely as a lever that pulls the SNAREs to trigger synchronous release [25]. While the Doc2B C_2_B domain binds to the SNARE complex through a different mode involving two helices (Figure 5B), modeling based on the rabphilin 3A‐SNAP‐25 crystal structure suggests that such binding also orients the C_2_B Ca^2+^‐binding loops away from the fusion site (Figure S16C). It is thus tempting to speculate that Doc2B also acts remotely upon Ca^2+^ binding to facilitate membrane fusion. We also note that the role of the Doc2B C_2_A domain is unclear at the moment, but its participation in membrane binding (Figure S11) suggests that it contributes to forming the Doc2B‐SNARE‐membrane assembly, and it is also plausible that it participates in Ca^2+^‐dependent rearrangements that trigger release.

Our data show that Cpx1 does not compete directly with Doc2B for binding to soluble SNARE complex (Figure S17) but there is partial disruption of Doc2B C_2_AB binding to the membrane‐anchored SNARE complex (Figure 5E), and Cpx1 enhances the ability of Syt1 C_2_AB to displace Doc2B C_2_AB from the SNARE complex (Figure 5D). These findings suggest that there are at least two types of primed SNARE complexes with distinct roles in evoked and spontaneous release, one type bound to Syt1 and complexin, and another type bound to Doc2.

### 3.3 Perspective

The working model of Figure 7 proposes a natural order of events: co‐translocation of Doc2B and Munc13‐1 to the plasma membrane (Figure 7A,B) should occur first such that both proteins can control exocytosis, whereas binding of Doc2B to the SNARE complex should occur late (Figure 7D), underlying a state that mediates spontaneous and perhaps other forms of release. The study presented here describes the structural basis for the Doc2B‐Munc13‐1 interaction, which is well‐established to be key for Doc2 function [50, 52, 57], and provides key information to understand the interplay among the distinct interactions involving Doc2s. Furthermore, this study uncovered a novel MID‐membrane interaction and revealed the SNARE complex binding site of Doc2B C_2_B. However, extensive research will be required to test the model of Figure 7 and the physiological relevance of the MID‐lipid and Doc2B C_2_B‐SNARE interactions, as well as to connect them with the steps that lead to distinct forms of release. In particular, it will be important to examine whether the MID‐lipid interactions facilitate the release of the Munc13‐1 inhibition by the MID to enable SNARE complex assembly (Figure 7B,C), and whether this mechanism cooperates with the putative weakening of MID‐Munc13‐1 binding by phosphorylation of MID by EphB2 [57]. It will also be critical to determine whether binding of Doc2B C_2_B to SNAP‐25 via the two helices (7C) stimulates vesicle priming and, downstream, is a key element of the primed state that is ready for spontaneous release (Figure 7D). An understanding of the last steps that lead to Doc2B‐dependent release will also require full elucidation of the binding mode between Doc2B and membrane‐anchored SNARE complex and of the structural rearrangements caused by Ca^2+^ binding to Doc2B, as well as further investigation of how complexins affect these interactions. It will also be important to determine whether Doc2A binds via the same mode, as the similarity between the helices of Doc2B and rabphilin 3A is less evident in Doc2A (Figure S15).

To mechanistically dissect the stimulatory and inhibitory functions of Doc2s, it will likewise be important to reconstitute fusion in assays that recapitulate the geometry of the system with single‐vesicle resolution [101, 102] and to structurally characterize at high resolution Doc2B‐Munc13 and SNARE interactions on membranes, where they might potentially form structured oligomeric states [103]. Ultimately, it will be critical to understand how these interactions are spatially and temporally organized at the presynaptic active zone. The model of Figure 7 and the structural insights described here provide key frameworks to achieve these goals.

## 4 Methods

### 4.1 FRET‐Based Lipid and Cis‐SNARE Complex Binding Assays

All lipids were purchased from Avanti Research. Liposomes were prepared similarly as previously described [104]. Briefly, chloroform stocks of lipids were combined and dried with a nitrogen stream in a 45°C water bath followed by vacuum desiccation overnight (see figure legends for lipid compositions). Lipids were solubilized in HEPES buffer containing 2% (wt/vol) β‐octylglucoside (βOG) and sonicated in a bath sonicator for 5 min twice. For SNARE complex‐containing liposomes, SNARE complexes were formed by incubating Syntaxin‐1 FL, synaptobrevin(29‐93), TMR‐labeled 76C‐SNAP‐25(11‐82), and SNAP‐25(141‐203) at 4°C overnight in 25 mM HEPES pH 7.4, 500 mM NaCl, and 2% βOG, and were added immediately after lipid sonication. The SNARE complex‐lipid mixture was incubated at room temperature for 25 min. To form liposomes, lipids were dialyzed with a 10 kDa‐cutoff cassette (Thermo Fisher Scientific, cat. no. 66383) at 4°C with the buffer used for FRET experiments (see below) supplemented with 2 g/L of Amberlite XAD‐2 detergent‐absorbing beads (Sigma Aldrich 10357) (0.5 L for 1 h, 0.5 L for 2 h, then 1 L overnight). For Rho‐liposomes, lipid concentration was measured by Rhodamine‐PE fluorescence comparing with a standard curve. For liposomes containing TMR‐labeled SNARE complex, concentration was determined from the TMR fluorescence after solubilization with 2% (wt/vol) βOG. For liposomes lacking any fluorophore, lipid concentration was determined by the Stewart method [105].

FRET experiments were performed with a PTI QM‐400 QuantaMaster spectrofluorometer (Photon Technology International) with a 100 µl sample volume. For all experiments except those using Indo‐1, a 495 nm long‐pass filter (Edmund Optics #32‐753) was used to minimize light scattering. The sample was excited at 480 nm and emission was detected from 500 to 620 nm with a 2 nm step size and 0.5 s integration time. FRET measurements were made in a buffer consisting of 25 mm HEPES pH 7.4, KCl at the concentration described in the figure legends, 1 mM TCEP, 0.5% glycerol, 0.2 mM ATP, 0.25 mM MgCl_2_, and 1.5 µM bovine serum albumin (to prevent adhesion of Doc2B to the cuvette). A fixed concentration of FRET donor was titrated with increasing concentrations of FRET acceptor. Each point in the titration was corrected for light scattering by subtracting the spectra of an analogous sample prepared with unlabeled FRET donor protein. Procedures used to analyze Doc2B C_2_AB binding to liposome‐anchored SNARE complex as a function of Ca^2+^ are described in the Supporting Information.

### 4.2 Nuclear Magnetic Resonance

All NMR spectra were acquired at 25°C on either 600 MHz or 800 MHz Agilent DD2 NMR spectrometers equipped with cold probes. NMR experiments with the ^15^N‐Doc2B C_2_ domain(s) including those for SNARE complex titrations and resonance assignments were performed in 20 mM HEPES pH 7.4, 100 mM KCl, 1 mM EDTA, 0.5 mM TCEP (1 mM for Figure S10), and 7% D2O. The soluble SNARE complex was assembled by mixing synaptobrevin(29‐93), syntaxin‐1(191‐253), SNAP‐25(11‐82), and SNAP‐25(141‐203), and free SNAREs were removed by concentration‐dilution with a 10 kDa cutoff as previously described [26]. NMR experiments with ^15^N‐Doc2B IDR were performed in 20 mM HEPES pH 7.4, 125 mM KCl, 1 mM EDTA, 0.5 mM TCEP, and 7% D2O. Resonance assignments for Doc2B C_2_A and Doc2B C_2_B^KKQQ^ were obtained using a set of standard double and triple‐resonance experiments, including ^1^H‐^15^N HSQC, HNCO, HNCACB, CBCA(CO)NH, (H)C(CO)NH‐TOCSY, and H(C)(CO)NH‐TOCSY [106].

### 4.3 Lipid‐ and Content‐Mixing Monitored Vesicle Fusion Assay

T‐Liposomes with encapsulated R‐phycoerythrin, biotin‐XX conjugate (1:500 P:L for syntaxin‐1; 1:400 P:L for dodecylated SNAP‐25) and V‐liposomes containing 1.5% NBD PE and 1.5% Marina Blue DHPE, as well as encapsulated Cy5‐streptavidin (1:500 P:L ratio for synaptobrevin) were prepared as previously described [7]. The fusion assays were adapted from [7]. Briefly, lipid and content mixing were monitored from the decrease in FRET between NBD and Marina Blue, and from the increase in FRET between Cy5 and R‐phycoerythrin, respectively, using a PTI QM‐400 QuantaMaster spectrofluorometer (Photon Technology International) equipped with a rapid Peltier temperature‐controlled four‐position sample holder at 37°C. T‐ and V‐ liposomes were mixed in the presence of 2.5 mM MgCl_2_, 2 mM ATP, 0.1 mM EGTA, 1 µM Munc18‐1, 200 nM Munc13‐1 C_1_C_2_BMUNC_2_C, 0.4 µM NSF, 2 µM αSNAP, 1 µM Doc2B (fragment, FL, or mutant), and 5 µm streptavidin at 37°C (200 µl total sample volume). After 10 min, 0.6 mM CaCl_2_ was added. Lipid mixing was normalized to the maximum signal for each sample after adding 1% β‐OG. Content mixing was normalized to the maximum signals obtained in control samples lacking streptavidin after addition of 1% β‐OG. Quantification of the amounts of lipid and content mixing was performed after 500 s in EGTA (Figure 6B,E and Figure S18B,E) such that substantial amounts of fusion had occurred, as fusion was generally inefficient under these conditions. Conversely, quantification of Ca^2+^‐dependent lipid and content mixing was performed at 11 min, shortly after the addition of Ca^2+^ (Figure 6C,F and Figure S18C,F), because fusion was highly efficient and differences among the results obtained for the different protein combinations were more overt than at the end of the assays, when fusion was complete for most protein combinations.

## Author Contributions

**Cyrus T. Rastegar**: conceptualization, investigation, writing – original draft, methodology, validation, visualization, formal analysis. **Julia Powell**: conceptualization, investigation, writing – review and editing, methodology, formal analysis. **Aleksandra Wosztyl**: investigation, methodology, software. **Guillaume David**: investigation, methodology, writing – review and editing, formal analysis. **Klaudia Jaczynska**: methodology, writing – review and editing, investigation. **Xiao‐chen Bai**: methodology, writing – review and editing, supervision. **Yan Han**: investigation, writing – original draft, methodology, visualization, validation, formal analysis, supervision. **Zhe Chen**: investigation, writing – review and editing, methodology, supervision. **Josep Rizo**: conceptualization, investigation, funding acquisition, writing – original draft, methodology, formal analysis, supervision, visualization.

## Funding

Grant RP220582 from the Cancer Prevention & Research Institute of Texas (CPRIT). Grant I‐1304 from the Welch Foundation (to JR). NIH R01 grant R01 NS150707 (to JR). NIH Research Project Award R35 NS097333 (to JR). NIH grant R24GM154185 to the Pacific Northwest Center for Cryo‐EM (PNCC).

## Conflicts of Interest

The authors declare no conflicts of interest.

## Supporting information




**Supporting File**: advs78011‐sup‐0001‐SuppMat.pdf.

## Data Availability

The initial cryo‐EM maps of the Doc2B‐Munc13‐1 complex without the designed binder and the locally refined map have been deposited in EMDB with accession codes EMD‐73826 and EMD‐73827, respectively. The final cryo‐EM map of the Doc2B‐Munc13‐1 complex aided by the designed binder has been deposited in EMDB with accession code EMD‐78200, and the corresponding raw movies are deposited in EMPIAR with accession code EMPIAR‐13857. The structural model of the Doc2B‐Munc13‐1 complex with the designed binder is deposited in the PDB with PDB accession code 37IC (Extended PDB ID pdb_000037IC). Several plasmids used in this study have been deposited in Addgene (see supplement). The NMR resonance assignments have been deposited in BioMagResBank with accession numbers 54031 and 54032. Other experimental data presented in this paper have been deposited in the Dryad database (DOI https://doi.org/10.5061/dryad.3r2280gwr).
