# Supplementary material for: Cryo‐EM and NMR Reveal a Membrane‐Gated Doc2B–Munc13‐1 Complex and a Helical Doc2B‐SNARE‐Binding Interface
Source: Adv Sci (Weinh). 2026 Nov 3:e78011. Online ahead of print. doi: 10.1002/advs.78011 (PMC13728869; doi:10.1002/advs.78011)
Supplement: Supplementary file 1 [file ADVS-9999-e78011-s001.pdf]

Supporting Information for

Cryo-EM and NMR reveal a membrane-gated Doc2B–Munc13-1 complex and a helical Doc2B-SNARE-binding interface

Cyrus T. Rastegar[1,2,3], Julia Powell[1], Aleksandra Wosztyl[1,2,3], Guillaume David[1,2,3], Klaudia Jaczynska[1,2,3], Xiao-chen Bai[1], Yan Han[1], Zhe Chen[1] and Josep Rizo[1,2,3]

[1] Department of Biophysics, University of Texas Southwestern Medical Center, Dallas, TX 75390, USA

[2] Department of Biochemistry, University of Texas Southwestern Medical Center, Dallas, TX 75390, USA

[3] Department of Pharmacology, University of Texas Southwestern Medical Center, Dallas, TX 75390, USA

Correspondence: Josep Rizo Jose.Rizo-Rey@UTSouthwestern.edu

This file includes:

Supplementary text
Supplementary Methods
Table S1
Figures S1 to S18
SI References

Supplementary text

To explain the decrease in FRET caused by Cpx1, it is important to consider the complex topology on a membrane. Syt1 C_2B binds to a membrane through its polybasic region while interacting with the SNAREs via the primary interface, with Cpx1 bound on the other side (1, 2) (Fig. S16A). In this orientation of the SNARE complex, Doc2B C_2B would project its polybasic region away from the plasma membrane (Fig. S16B). However, the NMR and FRET data (Fig. 4B, 4C, S13) suggest that Doc2B C_2AB binds to soluble SNARE complex with weaker affinity than to liposome-anchored SNARE complex and hence that binding of the polybasic region to the membrane cooperates with C_2AB binding to the SNARE complex, which thus would require a SNARE orientation distinct from that of Fig. S16B. Placing our model of the Doc2B C_2B-SNARE complex with the polybasic face bound to the membrane shows that this arrangement would lead to steric clashes of Cpx1 with the membrane (Fig. S16C). Clearly, the orientation of the Doc2B C_2B-SNARE complex is uncertain but Fig. S16C illustrates that, in this or similar configurations, Cpx1 binding would induce reorientation of the Doc2B C_2B-SNARE complex, which can explain the decrease in FRET caused by Cpx1 (Fig. 5E) and is expected to decrease the affinity of the Doc2B C_2B-SNARE complex, thus facilitating the replacement of Doc2B C_2B with Syt1 C_2B (Fig. 5D) to yield the configuration of Fig. S16A.

Supplementary methods

Recombinant protein expression and purification. Several new as well as previously undeposited plasmids created in our lab have been deposited in Addgene, and where applicable Addgene IDs are indicated in parentheses. Rat Munc13-1 constructs are numbered according to the NCBI Reference Sequence NM_022861.2. Munc13-1 contains a poorly conserved flexible loop spanning residues 1408-1452 which was deleted (Δ1408–1452) and in some cases replaced with EF. Purification of Munc13-1 MUN (residues 859-1516 Δ1408–1452 EF; Addgene #243818), $C_1C_2BMUNC_2C$ WT (residues 529-1735 Δ1408–1452 Δ1533-1551; Addgene #243821) or bearing the R1598E,F1658E mutations in C_2C (Addgene #243822) were

performed as previously described (3). SNAP-tag MUN (Addgene #243827) was generated by replacing the Δ1408–1452 EF in the MUN vector with SNAP-tag and was expressed and purified identically as MUN. The expression vector for full-length Chinese hamster NSF was a kind gift of Minglei Zhao (Addgene #102239) and purified as previously described (4). FL *Bos taurus* α-SNAP and full-length rat Munc18-1 were purified as in (5), and rat Synaptotagmin-1 C_2AB (residues 140-421) (6). FL SNAREs and Complexin-1 (Cpx1), and SNARE fragments were purified as per the following references: rat FL Syntaxin-1A (3) (1), rat FL synaptobrevin-2 (7), rat FL SNAP-25A (purification as in (8) and dodecylation as in (9)), rat FL Cpx1 (10), rat synaptobrevin-2 residues 29-93 (Syb29-93), rat Syntaxin-1A residues 191-253 (Syx191-253), human SNAP-25A residues 11-82 (WT and 76C) and 141-203, both with N-terminal tryptophans for concentration measurement, (SNAP-25(11-82) and SNAP-25(141-203)) (11).

All Doc2B constructs are based on NCBI Reference Sequence NP_112404.1. An expression vector for GST-thrombin-Doc2B C_2AB (residues 115-412) was a kind gift of Alexander Groffen (12). Expression vectors for Doc2B C_2A (residues 125-255; Addgene #243812) and C_2B (residues 265-412; Addgene #243811) were generated by inverse PCR. Doc2B C_2A K176Q,K179Q (Addgene #243824), Doc2B C_2B K316Q,K319Q (Addgene #243814), and Doc2B C_2AB K176Q,K179Q,K316Q,K319Q (Addgene #243828) were generated by site-directed mutagenesis. DNA encoding the Doc2B IDR was synthesized (Twist Biosciences) and cloned by Gibson assembly into a modified pGEX vector with a GST tag and TEV cut site to generate expression plasmids for GST-TEV site-Doc2B IDR (residues 1-124; Addgene #243817) or GST-TEV site-Doc2B FL (residues 1-412; Addgene #243813). A pGEX-4T-1 plasmid encoding the de novo designed Munc13 binder (Addgene #257809) was synthesized by Integrated DNA Technologies. The GST-TEV site-Munc13 binder- GGSGGSGGSGG-Doc2B IDR plasmid (Addgene #257810) was cloned by Gibson assembly. FL Doc2B F37A (Addgene #243823), FL Doc2B I20A,F37A, FL Doc2B K316Q,K319Q, and FL Doc2B K389Q were generated by site-directed mutagenesis.

For FRET experiments, an expression vector for a single cysteine mutant of Doc2B C_2AB (residues 126-412; G361C,C145A,C217A,C249A,C290A,C338A,C387A) was generously provided by Edwin Chapman (Addgene #134694). The G361C point mutation was reverted and an E266C mutation was introduced for site-specific labeling of the linker between C_2A and C_2B (C_2AB E266C; Addgene #243816), and the analogous vector for expression of Doc2B FL with a single cysteine at residue 266 (E266C) was generated by Gibson assembly (FL E266C; Addgene #243820) with IDR native cysteines also mutated to alanine. C_2AB E266C,K176Q,K179Q (#243825), K316Q,K319Q (#243826), and K389Q (#243829) mutants were generated by site-directed mutagenesis.

The de novo designed Munc13-1 binder protein was expressed in *Escherichia coli* BL21(DE3) cells with ampicillin at 37°C to OD = 0.8-1, and induced with 1 mM Isopropyl β-D-1-thiogalactopyranoside (IPTG) for 4 hours. The cell pellet was resuspended after centrifugation in phosphate buffered saline (PBS) with 2 mM DTT and protease inhibitor cocktail (Millipore Sigma P2714). Cells were lysed in an Avestin EmulsiFlex-C5 homogenizer, centrifuged at 20,000 rpm for 1 hr at 4 °C, then the supernatant was incubated with Glutathione Sepharose™ 4B resin for 2 hours at room temperature. The resin was washed sequentially with PBS + 2 mM DTT and PBS + 1 M NaCl + 2 mM DTT, followed by elution from the resin by thrombin cleavage in 50 mM Tris-HCl, pH 8.0, 150 mM NaCl, 2.5 mM $CaCl_2$, 0.5 mM TCEP for 3 hours at room temperature. Uncleaved protein was removed by re-passing through the Glutathione Sepharose™ 4B resin, followed by size exclusion chromatography with a Superdex 75 column (GE 16/60) in 20 mM HEPES pH 7.4, 100 mM KCl, 1 mM TCEP, 1 mM EGTA.

All Rat Doc2B constructs (including the de novo Munc13 binder – Doc2B IDR fusion protein) were expressed in Escherichia coli BL21(DE3) cells with ampicillin. Unlabeled proteins were expressed in LB broth while ^{15}N-labeled proteins were expressed in M9 minimal media with $^{15}NH_4Cl$ as the sole nitrogen source. For triple resonance experiments, ^{13}C-labeling was additionally achieved with $^{13}C_6$-glucose as the sole carbon source. Proteins were grown at 37°C to OD = 0.6-0.8, and induced with 0.4 mM IPTG at 20°C

for 16-20 hours. The bacterial pellet was harvested after centrifugation in 40 mM Tris-HCl pH 8.2, 1 M NaCl, 1% Triton X-100, 2 mM DTT, and protease inhibitor cocktail (Millipore Sigma P2714). Cells were lysed in an Avestin EmulsiFlex-C5 homogenizer, and after centrifugation at 20,000 rpm for 1 hr at 4°C the supernatant was incubated with protamine sulfate (0.1 g/L of culture) for 30 minutes at room temperature. The sample was centrifuged again at 20,000 rpm for 45 minutes, followed by incubation of the supernatant with Glutathione Sepharose™ 4B resin for 2 hours at room temperature. The resin was washed sequentially with 40 mM Tris-HCl pH 8.2 buffer containing 2 mM DTT and (A) 200 mM NaCl, (B) 200 mM NaCl + 50 mM $CaCl_2$, and (C) 1 M NaCl + 50 mM $CaCl_2$. Elution from the resin was achieved by TEV cleavage (in 50 mM Tris-HCl, pH 7, 150 mM NaCl, 0.5 mM EDTA, and 2 mM TCEP) or thrombin cleavage (50 mM Tris-HCl, pH 8.0, 150 mM NaCl, 2.5 mM CaCl2, 0.5 mM TCEP) at room temperature for 3 hr – 24 hr. Proteins were then re-passed on Glutathione Sepharose™ 4B resin to remove uncleaved protein, and further purified by cation exchange chromatography with a HiTrap® SP HP GE Healthcare column with buffer choice depending on the isoelectric point of the protein (for Doc2B FL and IDR constructs, the salt was always kept at or above 100 mM NaCl or KCl due to a strong tendency to precipitate at low ionic strength). All ion exchange buffers for constructs with C2 domains contained 5 mM $CaCl_2$ and only the Doc2-containing peak eluting at the highest salt concentration was taken to remove polyacidic contaminants. After ion exchange chromatography, proteins were further purified by size exclusion chromatography with a Superdex 75 column (GE 16/60) with buffer choice dependent on downstream application. Proteins were concentrated with an appropriate molecular-weight cut-off filter (MilliPoreSigmaTM AmiconTM UFC500324, UFC501024, or UFC503024) and used fresh or flash frozen with a protease inhibitor cocktail (1 µM antipain, 800 nM aprotinin, 20 µM leupeptin).

The LEA protein (RVLEAMShort; Addgene # 218122) expression and purification was adapted from ref. (13). Briefly, the LEA protein was expressed in Escherichia coli BL21(DE3) cells with ampicillin at 37 °C to OD = 0.8-1, cooled to 4 °C for 30 minutes, then induced with 0.2 mM IPTG overnight at 12 °C. Bacterial

pellets were centrifuged as described for the above proteins, and harvested in 20 mL/L of lysis buffer (50 mM HEPES pH 7.5, 300 mM NaCl, 10 mM imidazole, 1 mM TCEP and protease inhibitor cocktail (Millipore Sigma P2714)). Cells were lysed and supernatant collected as described for the above proteins, followed by incubation with 2mL slurry/L bacterial culture Thermo Scientific™ HisPur™ Ni-NTA Resin (Thermo Fisher 88222) for 30 minutes at room temperature. Resin was sequentially washed with (1) 10 CV lysis buffer, (2) 10 CV lysis buffer with 1M total NaCl, and (3) 10 CV lysis buffer, followed by elution with lysis buffer supplemented with 400 mM imidazole and further purification with a Superdex 75 column (GE 16/60).

BindCraft. BindCraft version 1.5.2 (14) was used to design a de novo designed binder against Munc13-1 with the following parameters: (1) binder length: 90-150 amino acids (2) Filters file: default_filters (3) Advanced file: default_4stage_multimer. The input pdb was generated from an AlphaFold3 (15) model of the Doc2B FL – Munc13 $C_1C_2BMUNC_2C$ complex. To improve computational speed, the model was truncated to include only Doc2B residues 11-21 and Munc13-1 residues 1161-1320. Note that this truncation exposed several buried hydrophobic residues of Munc13-1 which were mutated to lysine to reduce binder design against these artificially exposed hydrophobic patches.

Fluorophore conjugation. Single cysteine constructs of Doc2B and SNAP-25(11-82) were concentrated to 50-100 µM and labeled with 3-fold molar excess of either Alexa Fluor™ 488 C5 Maleimide (Thermo Fisher Scientific #A10254) or Tetramethylrhodamine-5-Maleimide (TMR) (Thermo Fisher Scientific #T6027) for 3 hours at RT or 4°C overnight in a 20 mM HEPES pH 7.4 buffer with 1 mM TCEP and 100-300 mM KCl. Labeling was quenched with 10 mM DTT for 10 minutes, and free dye was removed by size exclusion chromatography. SNAP-tag MUN at 50 µM was reacted with 75 µM SNAP-Surface® Alexa Fluor® 546 (NEB S9132S) in 25 mM HEPES pH 7.4 150 mM KCl, 2 mM TCEP for 2 hours at room temperature and free dye was removed by size exclusion chromatography.

Isothermal titration calorimetry. Soluble MUN domain, FL Doc2B, FL Doc2B F37A, the de novo Munc13-1 binder and the Munc13-1 binder – Doc2B IDR fusion protein were freshly purified for ITC, with the last purification step consisting of size exclusion chromatography with the ITC buffer (25 mM HEPES pH 7.4, 150 mM KCl, 1 mM EGTA, and 1 mM TCEP). Experiments were performed at 20°C in a MicroCal PEAQ-ITC (Malvern Panalytical). For titrations between MUN and FL Doc2B or FL Doc2B F37A, the cell contained 200 µl of ~20 µM Doc2B and the syringe ~200 µM of MUN and the experiment consisted of a single 0.5 µl injection followed by twenty 1.90 µl injections spaced 150 s apart. Triplicates of both sets of titrations were performed, integrated and baseline corrected in NITPIC (16), globally fit in SEDPHAT (17), and visualized in GUSSI (18). Titrations between MUN and the designed binder or the designed binder-Doc2B IDR fusion were performed and analyzed as above, except that the cell contained ~20 µM of MUN and ~200-300 µM of the binder or binder-Doc2B fusion protein. Note that the very high affinity of the binder-Doc2B fusion protein precluded reliable fitting to a kD.

FRET data analysis. Normalized FRET efficiency was calculated as follows:

$$(1)\ FRET = \frac{F_{donor} - F_i}{F_{donor}}$$

Where F_{donor} and F_i refers to the scatter-corrected fluorescence at 520 nm of the donor alone and at a given titration point, respectively. For competition experiments (Fig. 3E, 5D,E), fixed concentrations of both FRET donor and FRET acceptor were titrated with increasing concentrations of an unlabeled competitor. The % displacement (or % FRET decrease) of the acceptor from the donor was calculated as follows:

$$(2)\ \%\ Displacement = 100\ \% * \frac{F_i - F_{donor+acceptor}}{F_{donor} - F_{donor+acceptor}}$$

Where $F_{donor + acceptor}$ is the scatter-corrected fluorescence at 520 nm of a fixed concentration of donor and acceptor in the absence of competitor.

Ca^{2+} dependence of Doc2B C_2AB binding to liposome anchored SNARE complex. To examine binding of Alexa488-Doc2B C_2AB to liposome-anchored TMR-labeled SNARE complex as a function of Ca^{2+} concentration we used a ratiometric Ca^{2+} indicator, Indo-1 (19), which can be monitored independently of the Alexa488 and TMR dyes. The titrations used a commercial calcium calibration kit (Biotium #59100) consisting of buffer A (10 mM K2EGTA, 100 mM KCl and 10 mM MOPS, pH 7.20) and buffer B (10 mM CaEGTA, 100 mM KCl and 10 mM MOPS, pH 7.20). In all experiments, 25 µl of Indo-1, Doc2B C_2AB E266C, BSA (to minimize Doc2B C_2AB adhesion to the cuvette) and SNARE complex-liposomes (with residue 76 of SNAP-25(11-82) mutated to cysteine; 1:2000 P:L ratio; 38% POPC, 18% DOPS, 20% POPE, 20% cholesterol, 2% DAG, 2% PIP2) were dissolved in MOPS buffer (10 mM MOPS, 100 mM KCl, 0.5 mM TCEP, pH 7.2) and mixed with 75 µl of varying ratios of buffer A to buffer B to sample free Ca^{2+} concentrations between 0 and ~33.5 µM. The final samples consisted of 100 µl total volume with 7.5 mM EGTA and 250 nM Indo-1, 50 nM Doc2B, 1.5 µM BSA, and 900 nM SNARE complex-liposomes. Experiments were performed at 20 °C with temperature maintained with a rapid Peltier temperature-controlled sample holder using analogous procedures as the FRET binding experiments without Indo-1, except that the 495 nm longpass filter was omitted. The Alexa-488 emission scan was performed first, followed by an Indo-1 emission scan from 360 nm to 550 nm (excitation at 340 nm) with 2 nm step size and an integration time of 0.5s. Slit widths were set to 1.0 mm.

We first used the Ca^{2+} calibration kit to determine the Indo-1 – calcium K_D under the experimental conditions of the FRET assays. Specifically, in this calibration, the free Ca^{2+} concentrations for a given mixture of calibration buffer A to buffer B were calculated using a dissociation constant of EGTA for calcium of 150.5 nM (20). We first performed the aforementioned titration at these known free Ca^{2+} concentrations

with 250 nM Indo-1 and unlabeled Doc2B C_2AB and unlabeled SNARE complex-liposomes to mimic the conditions used for the FRET assays. The fluorescence emission spectra were blank-subtracted. The ratio R between the emission at 400 nm and 480 nm (F400/F480) was calculated at each point and fit to the Grynkiewicz equation (19):

$$(3)\ [Ca^{2+}] = K_d * \beta * \left(\frac{R - R_{min}}{R_{max} - R}\right)$$

Where K_d is the dissociation constant of Indo-1 for Ca^{2+}, R is the ratio of fluorescence at the Ca^{2+}-bound peak divided by fluorescence of the Ca^{2+}-free peak (F400/F480) for each titration point, Rmin is F400/F480 at zero Ca^{2+}, Rmax is F400/F480 at maximum Ca^{2+}, and β is F480 (zero Ca^{2+}) / F480 (maximum Ca^{2+}). The data was fit by non-linear least squares regression of the R-values against the calculated free Ca^{2+} concentrations with the Grynkiewicz equation (19) to Rmin, Rmax, and kD using SciPy v1.18.0 and NumPy v2.5.2 in Python v3.14.7. Data was visualized using Matplotlib v3.11.1. The calibrated Indo-1 dissociation constant for calcium (293 nM) was used to measure the free Ca^{2+} concentrations in the FRET titrations containing Indo-1, Alexa488-Doc2B C_2AB and TMR-SNARE complex-liposomes except for three points: at 100% buffer A and 100% buffer B, which are used to define Rmin and Rmax, respectively, for each titration, and for the 97.5% buffer B titration point, where the nominal free Ca^{2+} concentration from the EGTA – calcium dissociation constant was used since equation 3 becomes unreliable when approaching Rmax.

The apparent FRET values between Alexa488-Doc2B C_2AB and TMR-SNARE complex-liposomes as a function of Ca^{2+} concentration (Figure S7E, gray circles) were corrected for the scattering arising from the liposomes by subtracting spectra obtained with analogous samples prepared with unlabeled Doc2B C_2AB. To correct for the environmental sensitivity of Alexa-488, we performed an analogous titration with Indo-1 and Alexa488-Doc2B C_2AB but with unlabeled SNARE complex-liposomes. After subtracting for scattering (from an analogous titration but with unlabeled Doc2B C_2AB), any residual change in Alexa-488 fluorescence could be attributed to the dye environment. This change can be captured by a correction factor Q for each titration point by dividing the Alexa-488 fluorescence for each sample containing

Alexa488-Doc2B C_2AB and unlabeled SNARE complex-liposomes by the fluorescence of Alexa-488 in the absence of SNARE complex-liposomes. To obtain corrected FRET values for samples containing Alexa488-Doc2B C_2AB and TMR-SNARE complex-liposomes as a function of Ca^{2+} concentration (Figure S7E, black circles), the donor fluorescence (520 nm) at each point was then divided by corresponding Q to account for this environmental sensitivity.

Cryo-EM sample preparation and data acquisition. The initial cryo-EM sample containing only the FL Doc2B – Munc13 $C_1C_2BMUNC_2C$ complex was purified by size exclusion chromatography on a Superdex 200 Increase 10/300 GL column and flash frozen in liquid ethane at 0.8 mg/mL with a Vitrobot Mark IV (Thermo Fisher Scientific). Quantifoil R1.2/1.3 300 mesh Cu grids were glow-discharged for 80 seconds at -30 mA using the Pelco Easiglow. Approximately 3 μL of protein sample was added to the grid and blotted with the following settings: blot time = 2.5 s; wait time = 3 s; blot force = -15; relative humidity = 95%, temperature = 6°C. Initial grid screening was carried out at the UT Southwestern CryoEM Facility using a 200 kV Glacios microscope (Thermo Fisher Scientific), followed by a 24 hr data collection at the Pacific Northwest CryoEM Center (PNCC) on a 300 kV Titan Krios (Thermo Fisher Scientific) with EPU automated data collection. A total of 9,217 movies were collected in EER format with a defocus range of -2.2 to -0.9 μm, pixel size of 0.7296 Å, and total dose of ~50 e-/$Å^2$.

Analogous procedures were used to purify the complex for the second sample (containing the designed binder fused N-terminally to the IDR of Doc2B) and prepare the cryo-EM sample, except with 30 μM of RVLEAMShort added immediately before plunge-freezing. The dataset was also collected at PNCC on a 300 kV Titan Krios (Thermo Fisher Scientific). Two data collections (Data Collection #1 & #2) were performed on different microscopes. For Data Collection #1, a total of 6,147 movies were collected with serialEM in TIF format with a defocus range of -2.2 to -0.9 μm, pixel size of 0.4125 Å (super-resolution), and a total dose of ~60 e-/$Å^2$. For Data Collection #2, a total of 8,835 movies were collected with EPU

automated data collection in EER format with a defocus range of -1.0 to -3.0 µm, unbinned pixel size of 0.7277 Å, and total dose ~ 58 e-/Å^2.

Cryo-EM data processing. Cryo-EM data was processed using CryoSPARC (21) version 4.6 or higher. Beam-induced motion-correction and dose-weighting to compensate for radiation damage over spatial frequencies were performed using Patch Motion Correction in CryoSPARC. Contrast Transfer Function (CTF) parameters were estimated using Patch CTF estimation in CryoSPARC.

For the first sample, a total of 8,095 micrographs were selected for further processing from the total of 9,217 after manual curation. A total of 5,680,930 particles were extracted using the Blob picker algorithm during cryoSPARC live processing (Fig. S1B). Extracted particles (at 1.824Å/pixel) were subjected to two rounds of 2D classification, from which 702,437 particles were selected and re-extracted at 1.14Å/pixel. A subset of 69,402 particles was selected to generate an initial 3D model, followed by multiple rounds of 3D Heterogeneous Refinement, from which 282,997 particles were selected (Fig. S1B). These particles were then subjected to a Non-Uniform Refinement job, generating a reconstruction with an overall estimated resolution of 3.13Å (Fig. S1D).

To gain more insight into the putative interaction between Munc13 and Doc2B, a mask was generated near the region of the complex where extra densities were observed outside of the predicted Munc13 molecule. After particle subtraction followed by 3D classification (Fig. S1B, C), a clear density was observed in subclasses from the 282,997 particles (colored pink in Fig. S1C classes 2 and 3). 119,059 particles were then selected from these two classes and further local refinement in cryoSPARC generated a map with an overall reported resolution of 3.59Å (Fig. S1C, D). All resolutions were reported according to the gold-standard Fourier shell correlation (FSC) using the 0.143 criterion (22). Local resolution was estimated using CryoSPARC. Figures were generated using UCSF ChimeraX software (23). The cryo-EM

maps of the full complex and the locally refined map have been deposited in EMDB with accession codes EMD-73826 and EMD-73827, respectively.

Cryo-EM data processing for the second sample consisted of two sequentially processed data sets, with final particle stacks from each collection combined in the last refinement step (see Figure S3 and S4). Briefly, for Data Collection #1, 5,621 micrographs were initially selected after motion correction and CTF estimation within CryoSPARC. Particles were first selected using the Blob picker algorithm during cryoSPARC live processing. Next, 2D classification was performed and 2D classes with clear Munc13 features were selected and used as templates for further particle picking. A total of 2,912,470 particles were selected and extracted with a pixel size of 3.3 Å. A total of 852,916 particles were selected after one round of 2D classification. Next, three rounds of 3D classification were performed, the first two rounds used the 3D Heterogeneous job in CryoSPARC, whereas the last round was done in an Ab initio job with bin 2 particles (1.65 Å/pixel). Subsequently, these particles were re-extracted with the original pixel size of 0.825Å/pixel and subjected to a Non-Uniform Refinement job, generating a reconstruction with an overall estimated resolution of 3.46 Å (Fig. S3B). This volume was then used to create templates for another round of particle picking on both denoised (7,899,781 particles) and raw micrographs (7,851,941 particles) which were processed initially in parallel. The particle stack derived from the raw micrographs underwent 2D classification (711,918 particles selected), and two rounds of high-resolution ab-initio refinement (24) to yield a 133,272 particle stack. The particle stack derived from the denoised micrographs underwent one round of high-resolution ab-initio refinement to yield a 174,904 particle stack. This latter stack was used for 2D classification, from which a random subset of 5000 particles corresponding to end-on “top views” was used to train Topaz v0.2.5a (25) and pick 480,270 particles. These three particle stacks were then de-duplicated (604,851 particles remaining) and underwent a round of high-resolution ab-initio to yield a 298,700 particle stack. Duplicate particle removal followed by Non-Uniform refinement (1.13 Å/px with 280 px box) yielded a map at a reported resolution of 3.14 Å (291,350 particles). This map was used as a

template for particle picking on Data Collection #2, and the particles (288,838) were re-extracted at 0.825 Å/px with 404 px box to combine with the final particle stack of Data Collection #2.

For Data Collection #2, 8,835 micrographs underwent template picking on the raw (14,691,345 particles) and denoised micrographs (7,577,593 particles), which were combined, de-duplicated (14,097,585 particles remaining), and underwent 2D classification (812,829 particles selected), followed by one round of high-resolution ab-initio refinement (316,538 particles). These particles underwent 2D classification and a random subset of particles were used to train topaz on either end-on or side views, from which 1,462,836 and 2,133,716 particles were picked, respectively. These particle stacks underwent 2D classification yielding 452,236 putative "side view" particles and 752,162 "end-on view" particles. The two topaz-derived particle stacks and the 316,538 template-derived particle stack were de-duplicated and combined (1,237,094 particles remaining) followed by one round of high-resolution ab-initio refinement (532,428 particles). De-duplication and Non-Uniform refinement (1.27 Å/px with 256 px box) yielded a 3.05 Å resolution map (463,665 particles). Particles underwent global CTF refinement and were re-extracted at approximately 0.825 Å/px (0.82497 Å) in a 404 px box to match the pixel size of the first collection. The two particle stacks from Data Collection #1 and Data Collection #2 were combined in a final Non-Uniform refinement (0.82497 Å/px, 404 px box) to yield a map at 2.94 Å (728,080 particles). The cryo-EM map of the complex has been deposited in the EMDB with accession code EMD-78200; PDB accession code 37IC. The raw movies have been deposited to EMPIAR with accession code EMPIAR-13857 in two separate folders – "Movies_Collection1" and "Movies_Collection2" refer to movies corresponding to Data Collection #1 and Data Collection #2 in Figures S3 and S4, respectively.

Cryo-EM maps were post-processed using EMReady2 (26) (Fig. S4A). Refinement was performed against this map iteratively in Phenix (27) version 2.0-5936, Isolde version 1.12.0 (28) and Coot (29) version 1.3, starting from an AlphaFold (15) prediction of the structure of the complex between the binder-Doc2B

IDR fusion protein and Munc13-1 $C_1C_2BMUNC_2C$, with poorly-resolved regions removed and hydrogens added using ReadySet in Phenix.

Multiple sequence alignment. The following sequences were obtained from Uniprot: Rat Doc2B (P70610) Rat Rabphilin-3A (P47709) Rat Doc2c (F7ES77), Rat Doc2a (P70611), Rat Synaptotagmin-1 (P21707), and Rat Synaptotagmin-7 (Q62747), aligned with Clustal Omega (30), and visualized in Jalview version 2.11.5.0 (31).

Alphafold3. Structure predictions were performed with the AlphaFold Server (15).

Statistical analysis. Statistical analysis was performed with GraphPad Prism version 10.6.0 as described in the figure legends.

Table S1. CryoEM data collection and refinement statistics

	First dataset		Second dataset	
	Full complex (EMD-73826)	Focused map (EMD-73827)	Full complex (EMD-78200)	
**Data collection and processing**				
Magnification	165,000		105,000	165,000
Voltage (kV)	300		300	300
Electron exposure ($e^-/Å^{-2}$)	50		60	58
Defocus range (μm)	-0.9 to -2.2			-1.0 to -3.0
Pixel size (Å)	0.7296		0.825	0.7277
Symmetry imposed	C1			
Initial particle images (no.)	5,680,930		2,912,470	22,268,938
Final particle images (no.)	282,997	119,059	728,080	
Map resolution (Å)	3.13	3.59	2.94	
FSC threshold	0.143			
Map pixel size (Å)	1.14		0.82497	
**Refinement**				
Initial model used				AlphaFold Model
Model composition				
Nonhydrogen atoms				6,125
Number of Protein residues				754
Number of Ligands				0
R.m.s. deviations				
Bond lengths (Å)				0.004
Bond angles (°)				0.613
Validation				
MolProbity score				1.21
Clashscore				1.80
Poor rotamers (%)				0.44
Ramachandran plot				
Favored (%)				96.07
Allowed (%)				3.93
Disallowed (%)				0

Supplementary Figures

Figure S1. Cryo-EM supporting data for the first dataset. (A) A representative cryo-EM micrograph from data collection. The scale bar is 200 Å. (B) Cryo-EM data processing workflow. Representative 2D class averages are also shown. (C) Schematic of the focused classification and refinement workflow. Density of the bound Doc2B N-terminus in class 2 and 3 (colored pink) is discernable, and these two classes were selected for further local refinement. (D) Fourier shell correlation (FSC) of 3D map reconstructions. Top: full map as shown in panel B. Bottom: focused map as shown in panel C. (E) Orientation distribution of particles used for 3D map reconstructions. Top: full map as shown in panel in B. Bottom: focused map as shown in panel in C.

Figure S2. ITC analysis of binding of the de novo designed Munc13-1 binder (A) or the de novo binder-Doc2B-IDR fusion (B) to the Munc13-1 MUN domain. From global fits of three replicates, the Munc13-1 binder K_D [95% confidence level] = 328 nM [132, 721]; ΔH = -4.2 kcal/mol [-4.7, -3.8]. The fusion protein bound too tightly to be reliably fit and a representative titration is shown.

Figure S3. Cryo-EM supporting data for the second dataset, consisting of two separate 24-hour collections of the same sample grid on two different microscopes (labeled Data Collection #1 and Data Collection #2). (A) A representative cryo-EM micrograph from data collection. The scale bar is 200 Å. (B) Cryo-EM data processing workflow for Data Collection #1. Representative 2D class averages are also shown.

Figure S4. Cryo-EM supporting data for the second dataset, consisting of two separate 24-hour collections of the same sample grid on two different microscopes (labeled Data Collection #1 and Data Collection #2).

(A) Cryo-EM data processing workflow for Data Collection #2. Representative 2D class averages are also shown. Note that the best map from Data Collection #1 was used to create templates for the initial particle picking in Data Collection #2. Cleaned particle stacks from both collections were combined for the final refinement and post-processed with EMReady2. (B) Local resolution estimation of the unsharpened map in the same orientation as that of Figure 1B to allow ready comparison and identification of the Doc2B MID surface. (C) Gold-standard Fourier shell correlation (FSC) of the final 3D map reconstruction. The reported resolution of 2.94 Å corresponds to the corrected curve at FSC = 0.143 (D) Orientation distribution of particles used for the 3D map reconstructions (log scale). (E) Conical FSC summary plot. Conical FSC area ratio (cFAR) = 0.60 (3072 conical directions; 20 ° cone half-angle) with directional resolution ranging from 2.67 Å to 3.15 Å. (F) Fourier sampling distribution evaluated at a Fourier radius of 113 (2.95 Å). Fourier space was completely sampled (0.0% empty bins) with Sampling Compensation Factor (SCF) = 0.979.

Figure S5. An F37A mutation impairs binding of Doc2B to Munc13-1. (A,B) ITC analysis of binding of FL Doc2B WT (A) or F37A mutant (B) to the Munc13-1 MUN domain. Doc2B WT K_D [95% confidence level] = 153 nM [52, 375]; ΔH = -7.0 kcal/mol [-7.8, -6.4]. Doc2B F37A K_D = 1.42 µM [0.71, 2.89]; ΔH = -6.7 kcal/mol [-8.8, -5.6]. K_Ds and enthalpies are from global fits of three replicates.

Figure S6. The Munc13-1 MUN domain competes with lipids for binding to FL Doc2B but not Doc2B C_2AB. (*A*-*C*) FRET experiments analogous to those of Figure 3D but with 10-fold higher liposome concentration (1 mM lipids) such that a large fraction of both FL Doc2B and Doc2B C_2AB are lipid-bound (*A*-*B*). The plots show representative spectra of 50 nM Alexa-488-labeled FL Doc2B (*A*) or Doc2B C_2AB (*B*) alone (blue curves) or bound to 1000 µM lipids in the absence (red curves) or presence of the indicated Munc13-1 MUN domain concentrations. (*C*) Percentage of displacement of FL Doc2B (black) or Doc2B C_2AB (red) from

the liposomes (1000 µM lipids) as a function of MUN concentration for n = 3 replicates. Error bars are SDs. (D-F) FRET experiments of 50 nM Alexa488-FL Doc2B with 150 nM Alexa546-SNAP-MUN and varying liposome concentrations. (D) Design of the FRET assay. (E) Representative spectra of 50 nM FL Doc2B alone (blue) or with 150 nM MUN (red) and with the addition of either 500 (green) or 1000 µM (black) unlabeled liposomes. (F) Plot of the percentage of Alexa546-SNAP-MUN displacement from Alexa488-FL Doc2B as a function of liposome concentration (expressed as total lipid concentration) for n = 3 replicates. Error bars are SEMs.

Figure S7. Analysis of the Ca^{2+} dependence of binding of Doc2B C_2AB to liposome-anchored SNARE complex. (*A*) Representative fluorescence spectra of 50 nM Alexa-488 Doc2B C_2AB in the presence of 0.5 mM Ca^{2+} and the absence (black) or presence (red) of 500 nM liposome-anchored TMR-labeled SNARE complex (1:2000 P:L ratio). (*B*) Bar diagram showing the average of the fluorescence intensity at 520 nm for three replicates of the experiments performed under the conditions of (*A*). Experiments were performed in 100 mM KCl at pH 7.4. Liposomes were composed of 38% POPC, 18% DOPS, 20% POPE, 20% Cholesterol, 2% PIP2, and 2% DAG. (C) Emission spectra of Indo-1 (excitation at 340 nm) at the free Ca^{2+} concentrations indicated by the color code to calibrate the dye. Unlabeled Doc2B C_2AB (50 nM) and unlabeled SNARE complex-liposomes (900 nM) were included to mimic the conditions used for the FRET assays. Note the isoemissive point at approximately 458 nm. The ratio R was calculated at F400 and F480 (dashed vertical lines). (D) The R values obtained from the data in panel (C) (black dots) were fit (red line) to the Grynkiewicz equation to Rmin, Rmax, and kD. Dashed lines indicate Rmin and Rmax from the fit, and the error shown for kD refers to the standard error of the fit. The K_d derived from these data was 293 ± 6 nM. The calibration was performed under analogous conditions as the experiments of panels (E, F). (E-F) Ca^{2+} titration of Doc2B and SNARE complex-liposomes with free Ca^{2+} ranging from 0 to ~33.5 µM. The final Indo-1, Doc2B C_2AB, and SNARE complex-liposome concentrations were 250 nM, 50 nM, and 900 nM,

respectively, and where applicable Doc2B C_2AB was labeled with Alexa-488 and SNARE complex-liposomes with TMR. All experiments were performed at 20 °C. In (E), the apparent FRET between Alexa488-Doc2B C_2AB and TMR-SNARE complex (gray circles) was monitored as a function of Ca^{2+}. Scattering arising from the liposomes was subtracted using spectra obtained with analogous samples prepared with unlabeled Doc2B C_2AB. An increase in apparent FRET was observed from nM to low single-digit μM Ca^{2+} concentrations, which was followed by a sharp decrease in FRET at higher free Ca^{2+} concentrations. Because the apparent FRET became negative, we hypothesized that a change in environment of the Alexa-488 probe induced by Ca^{2+} binding to Alexa488-Doc2B C_2AB caused an increase in its fluorescence. To test this hypothesis, we performed a Ca^{2+} titration with samples that were analogous to those used for the data of panel (E) but using Alexa488-Doc2B C_2AB and unlabeled SNARE complex-liposomes. We indeed observed a Ca^{2+}-dependent increase in fluorescence and for each Ca^{2+} concentration we calculated a Q factor defined as the ratio between the fluorescence of Alexa488-Doc2B C_2AB observed in these experiments (performed with unlabeled SNARE complex-liposomes) by the fluorescence of Alexa488-Doc2B C_2AB in the absence of liposomes (F). We then used this Q factor to correct the apparent FRET of the experiments of panel (E) and derive corrected FRET values (black circles). Free Ca^{2+} concentrations were calculated from the Indo-1 fluorescence in the same sample using the Indo-1 – Ca^{2+} K_d obtained from the calibration of (C,D) except for the zero and maximum Ca^{2+} points, which were used to define Rmin and Rmax, respectively, and for the second highest Ca^{2+} concentration, where the nominal value from the added EGTA and Ca^{2+} concentrations was used because Indo-1 was too saturated to reliably apply the Grynkiewicz equation for this point. The bars in (E) are standard deviations calculated from two separate titrations.

Figure S8. ^1H-^{15}N TROSY-HSQC spectra of 50 μM Doc2B C_2AB K316Q,K319Q in the absence (black contours) or presence of the indicated SNARE complex concentrations (cyan and red contours).

Figure S9. Analysis of Doc2B C_2A domain binding to the SNARE complex by NMR spectroscopy. (*A*) ^1H-^{15}N TROSY-HSQC spectra of 67 μM Doc2B C_2A domain in the absence (black contours) or presence of the indicated SNARE complex concentrations (cyan, red and green contours). Contours were adjusted to allow observations of most cross-peaks from each spectrum and illustrate the multiple cross-peak shifts observed. (*B*) ^1H-^{15}N TROSY-HSQC spectrum of Doc2B C_2A domain showing the cross-peak assignments with the corresponding residue numbers. The cross-peaks of residues 126 and 177 are indicated with a * because they are not observable at the contour levels chosen to facilitate cross-peak dissection in the crowded regions of the spectrum. (*C, D*) Ribbon diagrams of the Doc2B C_2A domain (PDB accession code 4LCV) with residues corresponding to cross-peaks that shift substantially upon binding to the SNARE complex in orange (*C*) ($\Delta\delta^1H > 0.02$ ppm and/or $\Delta\delta^{15}N > 0.2$ ppm) or with acidic and basic residues colored in red and blue, respectively (*D*).

Figure S10. ^1H-^{15}N TROSY-HSQC spectra of 50 μM Doc2B C_2B K316Q,K319Q in the absence (black contours) or presence of the indicated SNARE complex concentrations (cyan and red contours). Contours were adjusted to allow observations of all cross-peaks from each spectrum and illustrate the absence of clear cross-peak shifts.

Figure S11. Mutations in the polybasic regions of the Doc2B C_2A and C_2B domains impair membrane binding. (*A*-*C*) Ribbon diagrams of the Syt1 C_2B domain (*A*), the Doc2B C_2A domain (*B*) and C_2B domain (C) (PDB accession codes 1K5W, 4LCV and 4LDC, respectively). Colored in yellow are R322 and K325 for Syt1 C_2B, which were mutated in previous studies (32), K176 and K179 for Doc2B C_2A and K316 and K319 for Doc2B C_2B. (*D*) FRET between Alexa-488-labeled WT (blue circles) or K176Q,K179Q (red squares) or K316Q,K319Q (green triangles) Doc2B C_2AB and Rho-liposomes as a function of lipid concentration. The

KCl concentration was lowered to 50 mM KCl to strengthen the binding interaction, allowing quantitative comparisons of binding affinity between WT and polybasic mutants. Each point is from a representative titration and the solid curves are global fits of three replicates with a Hill equation to a shared kD. Calculated K_Ds [95% confidence level]: WT, 65 µM [63, 67]; K176Q,K179Q: 301 µM [285, 320]; K316Q,K319Q: 197 µM [191, 204]. Liposomes were composed of 38% POPC, 18% DOPS, 15% POPE, 20% Cholesterol, 5% Rhodamine-PE, 2% PIP2, and 2% DAG.

Figure S12. ^{1}H-^{15}N TROSY-HSQC spectra of 70 µM Doc2B C_2A K176Q,K179Q mutant in the absence (black contours) or presence of the indicated SNARE complex concentrations (cyan, red and green contours). Note that only very small perturbations of a few cross-peaks are observed even at 80 µM SNARE complex and that such small changes can readily be caused by very weak, non-specific interactions due to the high sensitivity of the cross-peak chemical shifts and intensities to such interactions.

Figure S13. ^{1}H-^{15}N TROSY-HSQC spectra of 70 µM Doc2B C_2AB K176Q,K179Q,K316Q,K319Q in the absence (black contours) or presence of the indicated SNARE complex concentrations (cyan, red, green and magenta contours).

Figure S14. ^{1}H-^{15}N TROSY- HSQC spectrum of Doc2B C_2B K316Q,K319Q showing the cross-peak assignments with the corresponding residue numbers.

Figure S15. Multiple sequence alignment of the two helices at the tip of the C_2B domain β-sandwich implicated in SNARE binding including the rat isoforms of the following proteins (Uniprot ID in parenthesis): rabphilin 3A (P47709), Doc2B (P70610), Doc2C (F7ES77), Doc2A (P70611), Syt1 (P21707), and Syt7 (Q62747). Residues from rabphilin 3A that contact SNAP-25 in the crystal structure of their

complex (33) are indicated with *. Note the high degree of similarity between Doc2B and Rph3A compared to the other Doc2 isoforms and Synaptotagmins.

Figure S16. Model of how Cpx1 may distort the binding mode between the Doc2B C_2B domain and membrane-anchored SNARE complex. The diagrams show models of membrane-anchored SNARE complex bound to Cpx1(26-83) and Syt1 C_2B (*A*), to Doc2B C_2B, Cpx1(26-83) and Syt1 C_2B (B) and to Cpx1(26-83) and Doc2B C_2B domain with its polybasic region bound to the membrane (C) (lipids in gray). The configurations were built superimposing the crystal structures of the SNARE complex bound to Syt1 C_2B (1), the Cpx1(26-83) fragment (34) and our model of the Doc2B C_2B-SNARE complex (Fig. 5F), which is based on the crystal structure of the rabphilin 3A C_2B-SNAP-25 complex (33) and our NMR data showing that the same two helices of rabphilin 3A that bind to SNAP-25 in this structure are involved in binding of the Doc2B C_2B domain to the SNARE complex. The superposition of (C) is the same shown in Fig. 5G without the membrane. The orientation of the Syt1 C_2B domain with respect to the membrane in (A,B) is based on all-atom MD simulations (2) and is supported by the effects of mutations in the polybasic region on membrane binding (11). The orientation of the Doc2B C_2B domain with respect to the membrane was built manually in Pymol assuming that it is similar to that adopted by Syt1 C_2B and is supported by the effects of mutations in the Doc2B C_2B polybasic region on liposome binding (Fig. S11). Cyan spheres show the Ca^{2+}-binding sites of the Syt1 and Doc2B C_2B domains to indicate their locations, but note that all the interactions shown in the panels occur in the absence of Ca^{2+}. N and C indicate the N- and C-termini of the SNARE motifs. Note that the model of (C) predicts that the Doc2B C_2B domain Ca^{2+}-binding loops point away from the SNARE complex C-terminus that induces membrane fusion.

Figure S17. Cpx1 does not compete with Doc2B $C_2AB^{KKQQ,KKQQ}$ for binding to the SNARE complex. (A) ^1H-^{15}N TROSY-HSQC spectra of 70 μM Doc2B C_2AB K176Q,K179Q,K316Q,K319Q in the presence of 80 μM SNARE

complex without (black contours) or with (red contours) 90 μM Cpx1. (B) Ratios of the intensities of the ^{1}H-^{15}N TROSY-HSQC cross-peaks from $C_2AB^{KKQQ,KKQQ}$ bound to the SNARE complex without (I_{SC}) or with bound Cpx1 ($I_{SC+Cpx1}$) with respect to the intensities of $C_2AB^{KKQQ,KKQQ}$ alone (I_{-SC}). Cross-peak numbers in the x axis were arbitrarily assigned by the automated peak picking algorithm of NMRViewJ. The similarity of the ratios shows that the SNARE complex induces similar broadening of the $C_2AB^{KKQQ,KKQQ}$ cross-peaks in the absence and presence of Cpx1, showing that Cpx1 does not displace $C_2AB^{KKQQ,KKQQ}$ from the SNARE complex. Note that ratios around 1 correspond to cross-peaks from residues that are unstructured and remain flexible upon SNARE complex binding.

Figure S18. Modulation of SNARE and Munc13-1-dependent liposome fusion by Doc2B. (*A, D*) Lipid mixing between V- and T-liposomes was measured from the fluorescence de-quenching of Marina Blue-labeled lipids in the V-liposomes. These curves were obtained simultaneously with the content mixing curves of Fig. 6A,D. Assays were performed in the presence of Munc18-1, NSF, αSNAP and Munc13-1 $C_1C_2BMUNC_2C$ in the absence (T + V) or presence of FL Doc2B or the indicated Doc2B fragments. '− Munc13' refers to a negative control analogous to T + V but with Munc13-1 omitted. Experiments were started in the presence of 100 μM EGTA and 5 μM streptavidin, and Ca^{2+} (600 μM) was added after 600 s. (*B, C, E, F*) Quantification of the lipid mixing observed in EGTA after 500 s (*B, E*) and in Ca^{2+} after 11 min (*C, F*). Error bars are standard deviations. Statistical significance and p values with respect to T+V (*B, C*) or WT FL Doc2B (*E, F*) were determined by ordinary one-way ANOVA with Holm-Sidak's multiple comparisons test (ns = not significant; * $p \leq 0.05$; ** $p \leq 0.01$; *** $p \leq 0.001$; **** $p \leq 0.0001$).

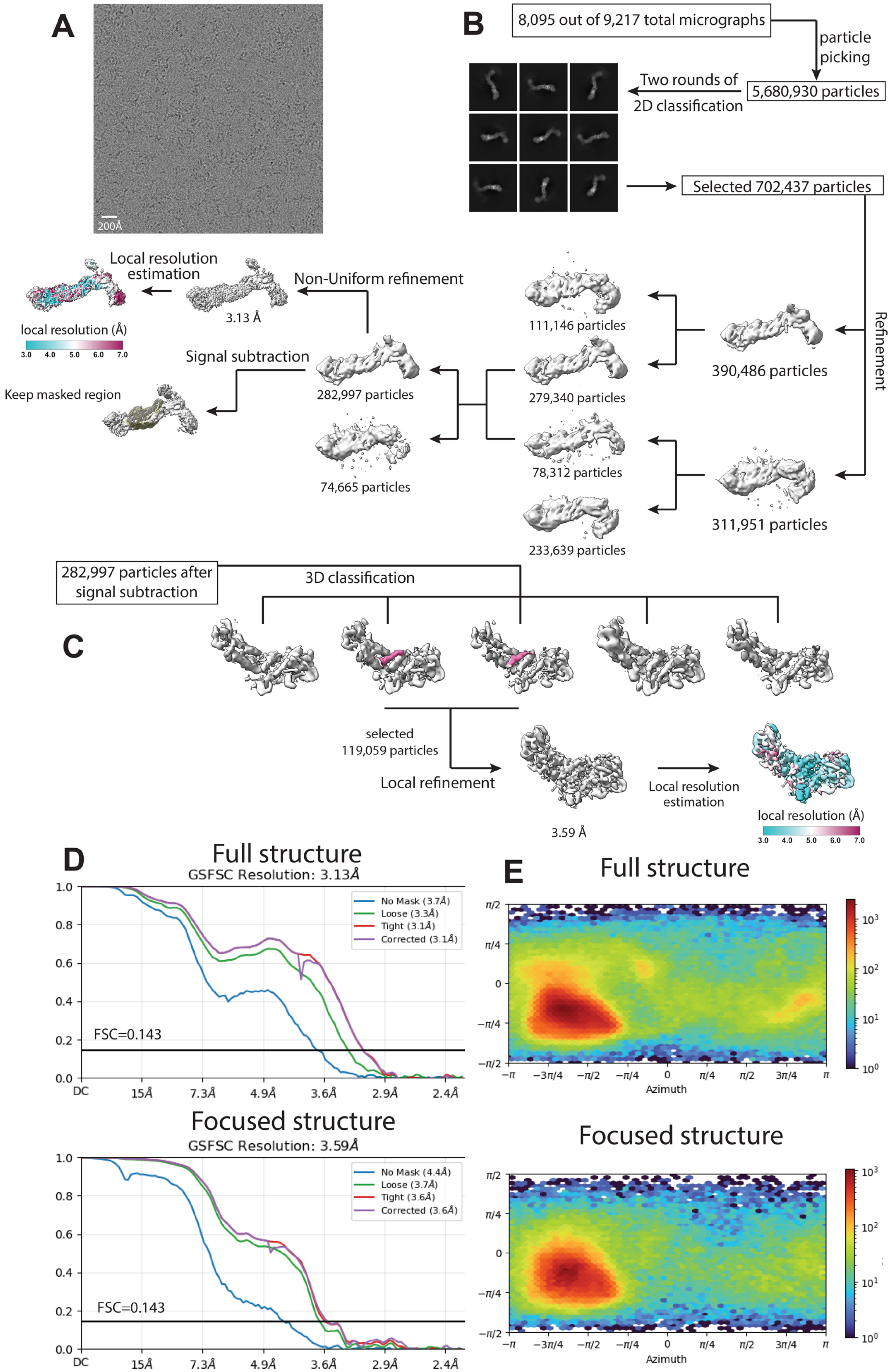

Fig. S1
Rastegar et al.

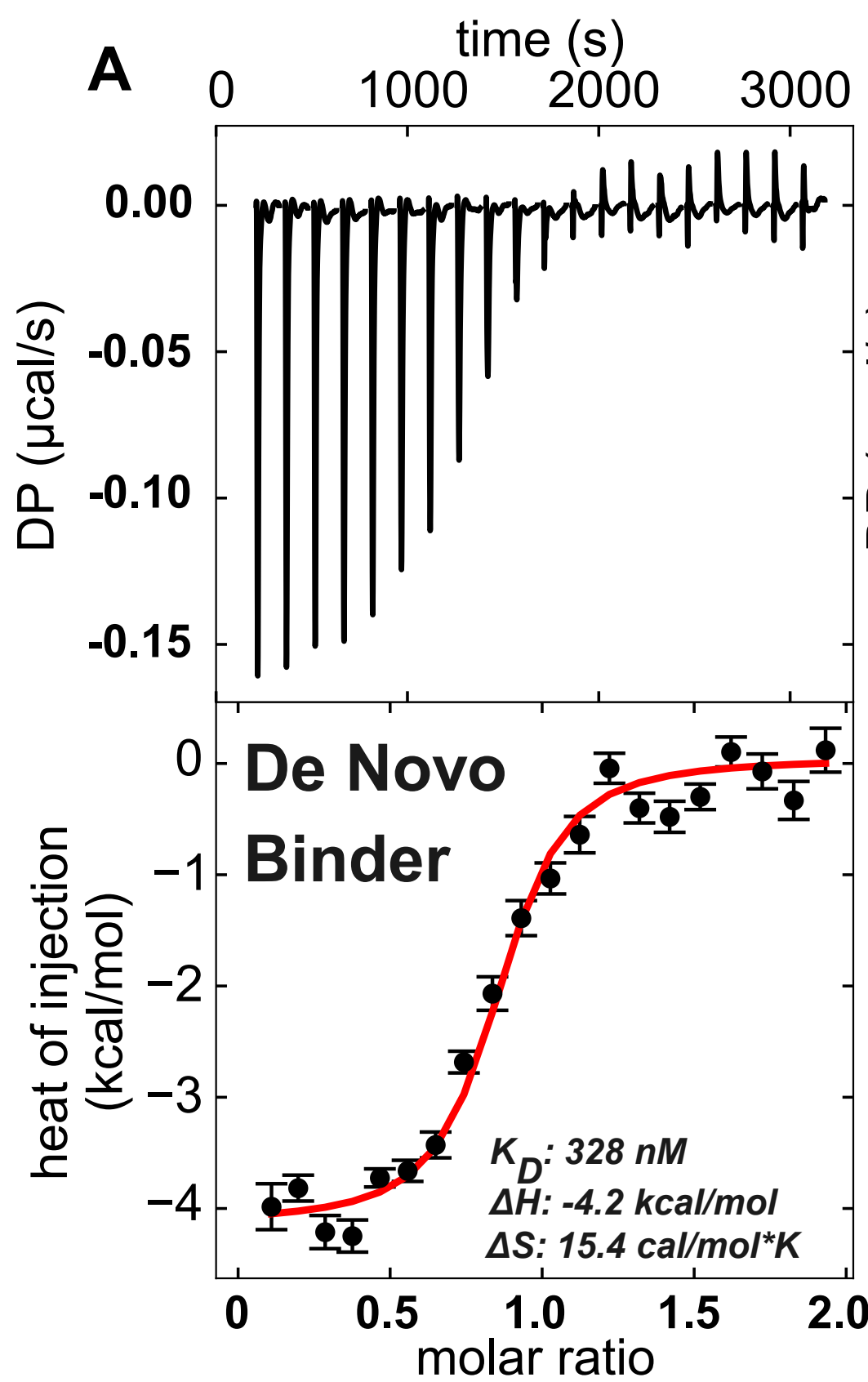

Rastegar et al.
Figure S2

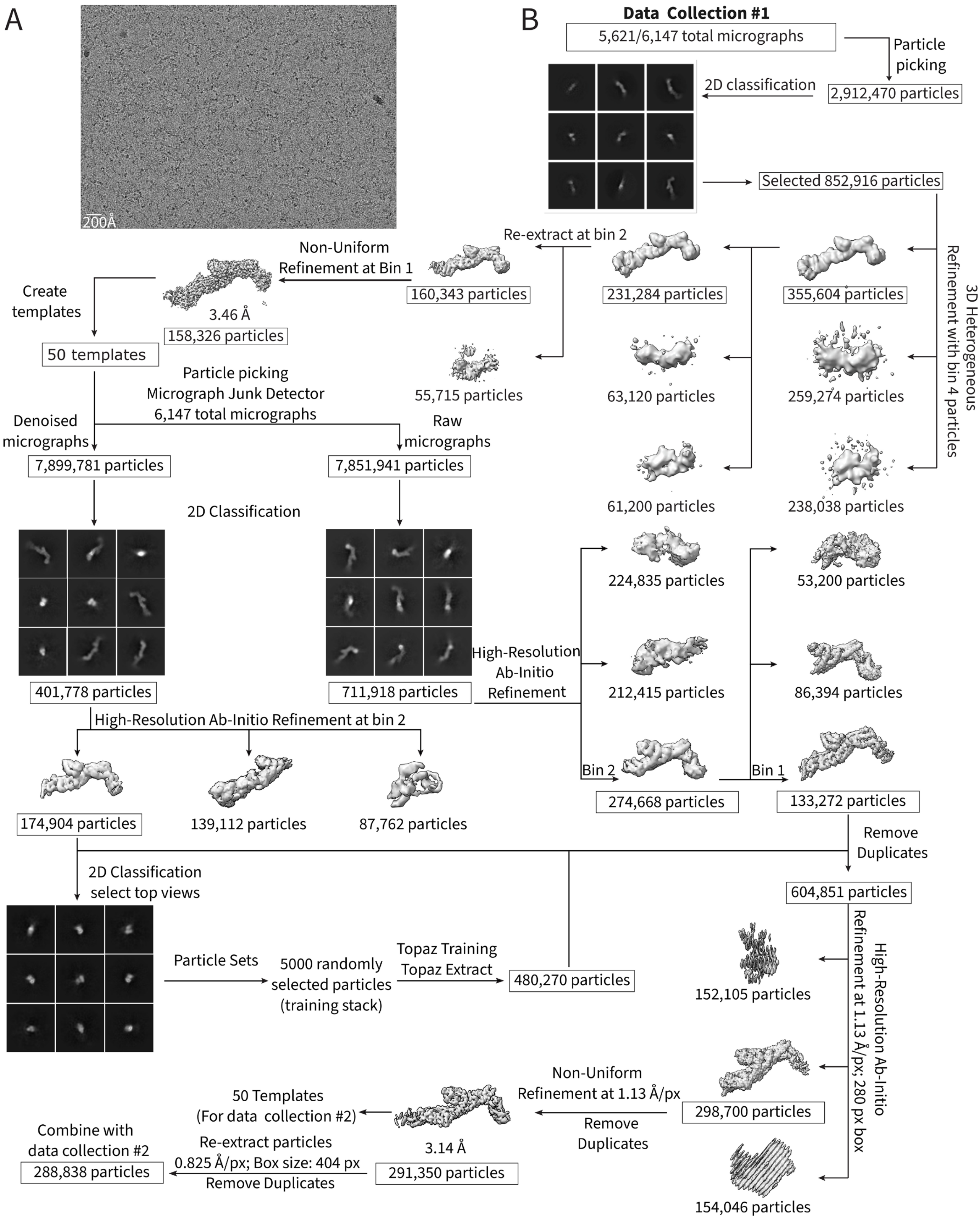

Rastegar et al.
Figure S3

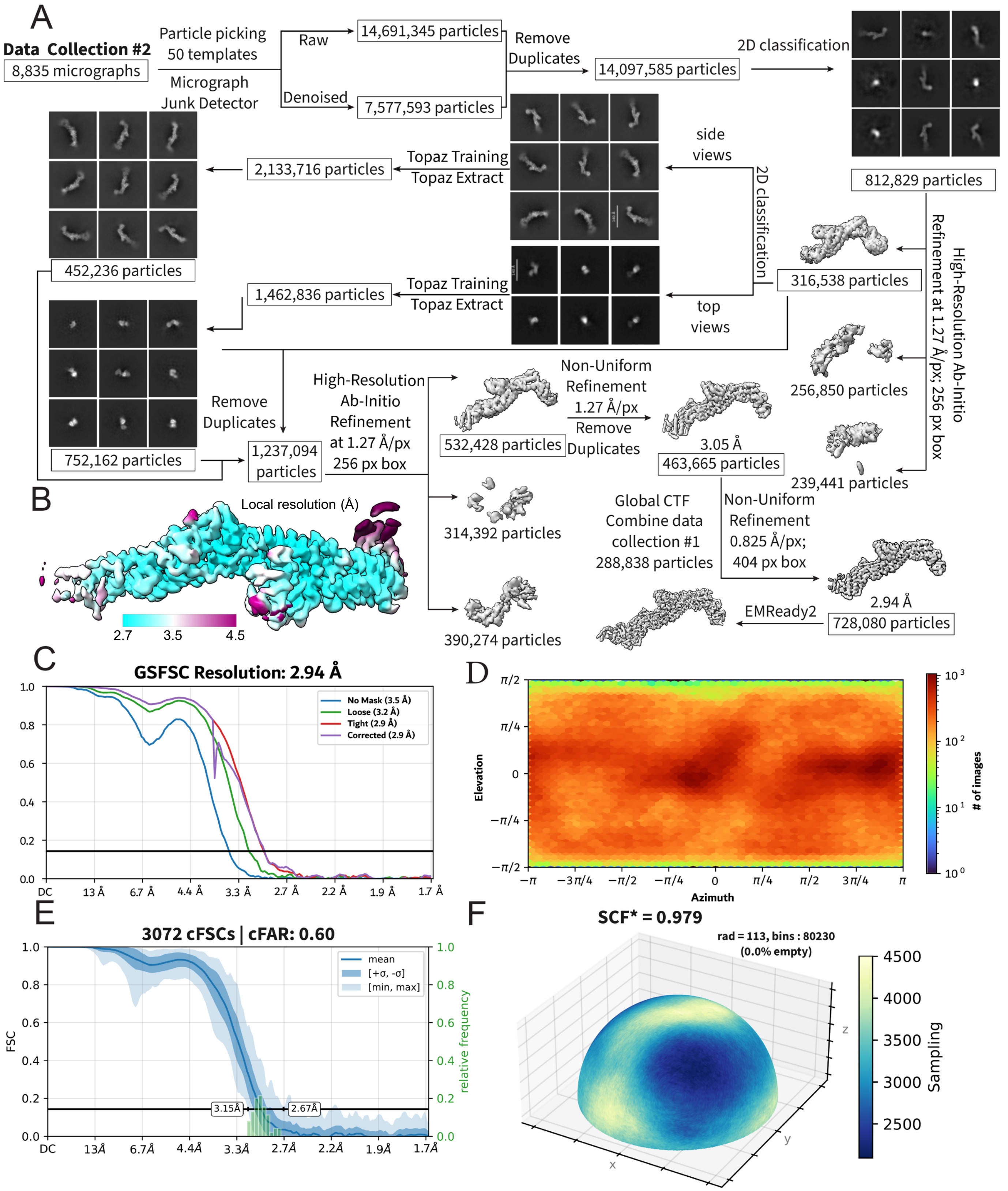

Rastegar et al.
Figure S4

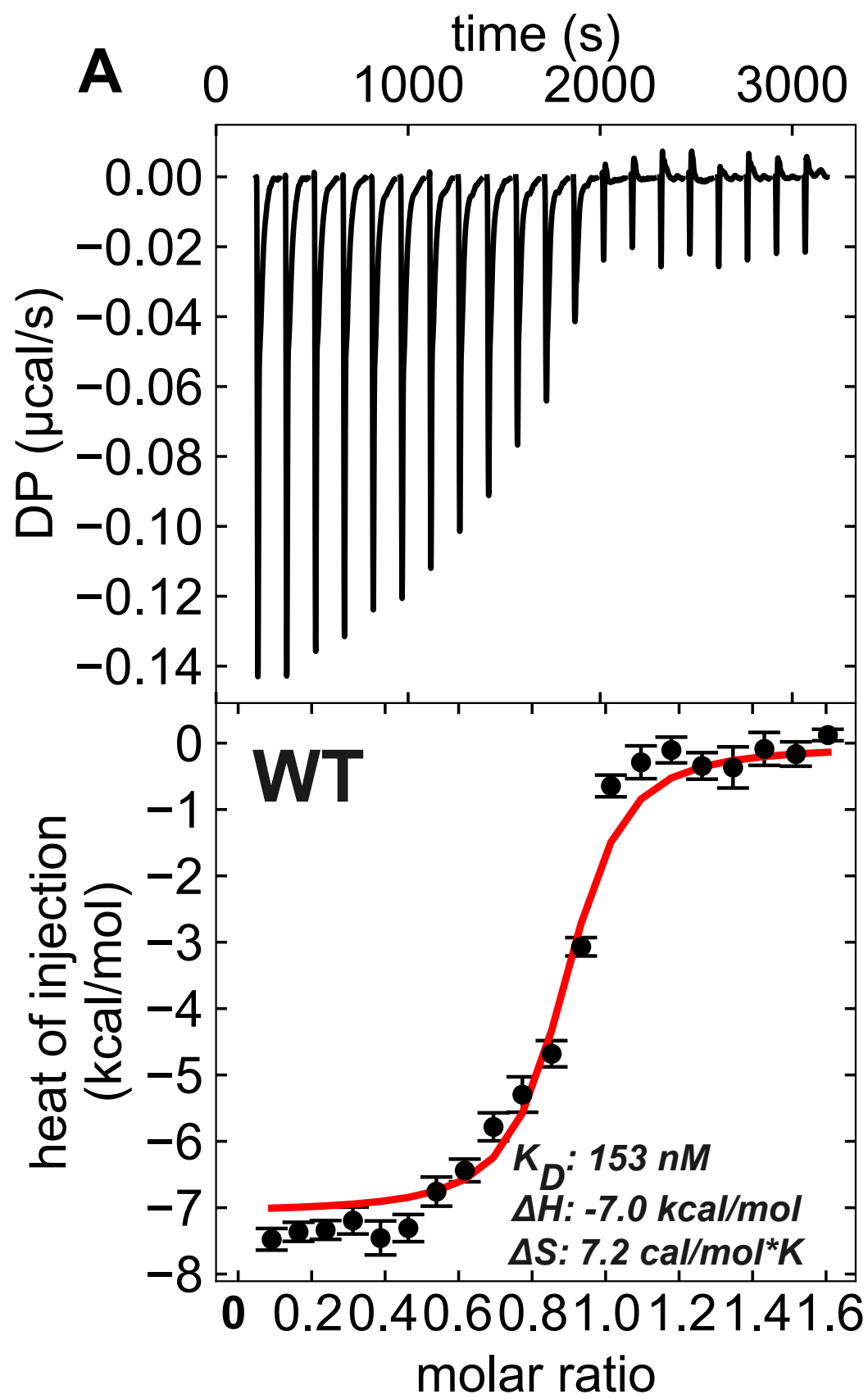

Rastegar et al.
Figure S5

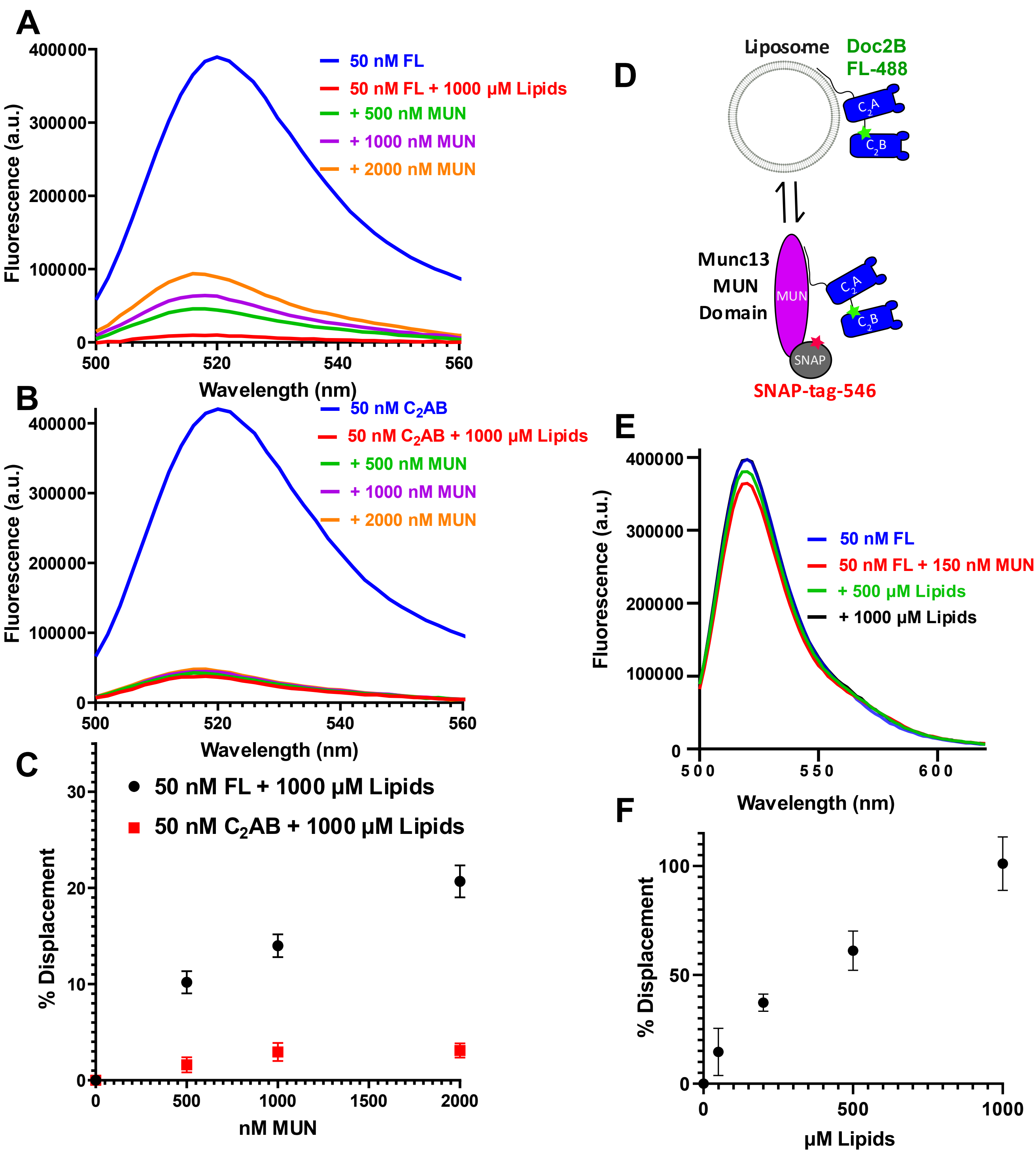

Rastegar et al.
Figure S6

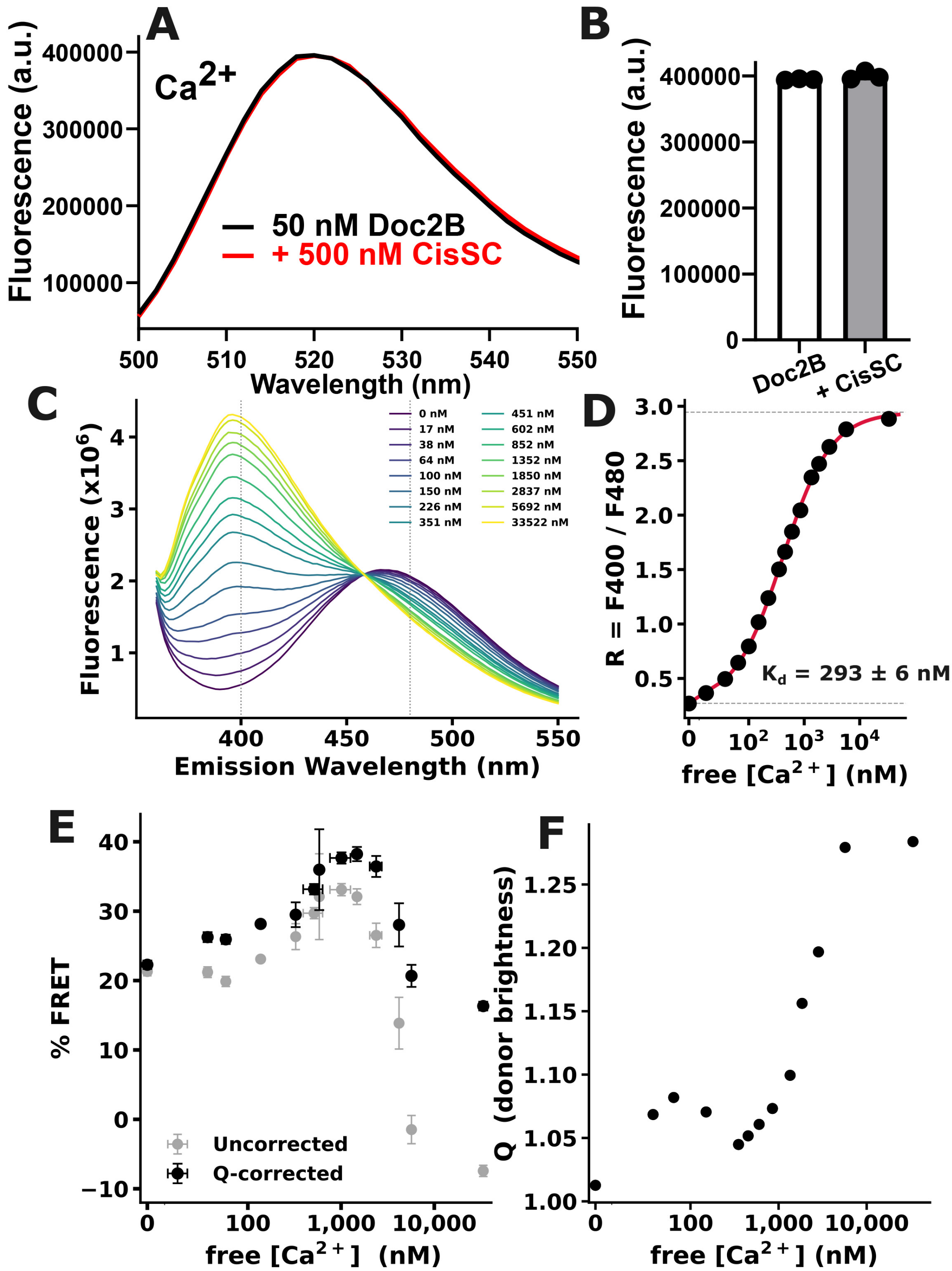

Rastegar et al.
Figure S7

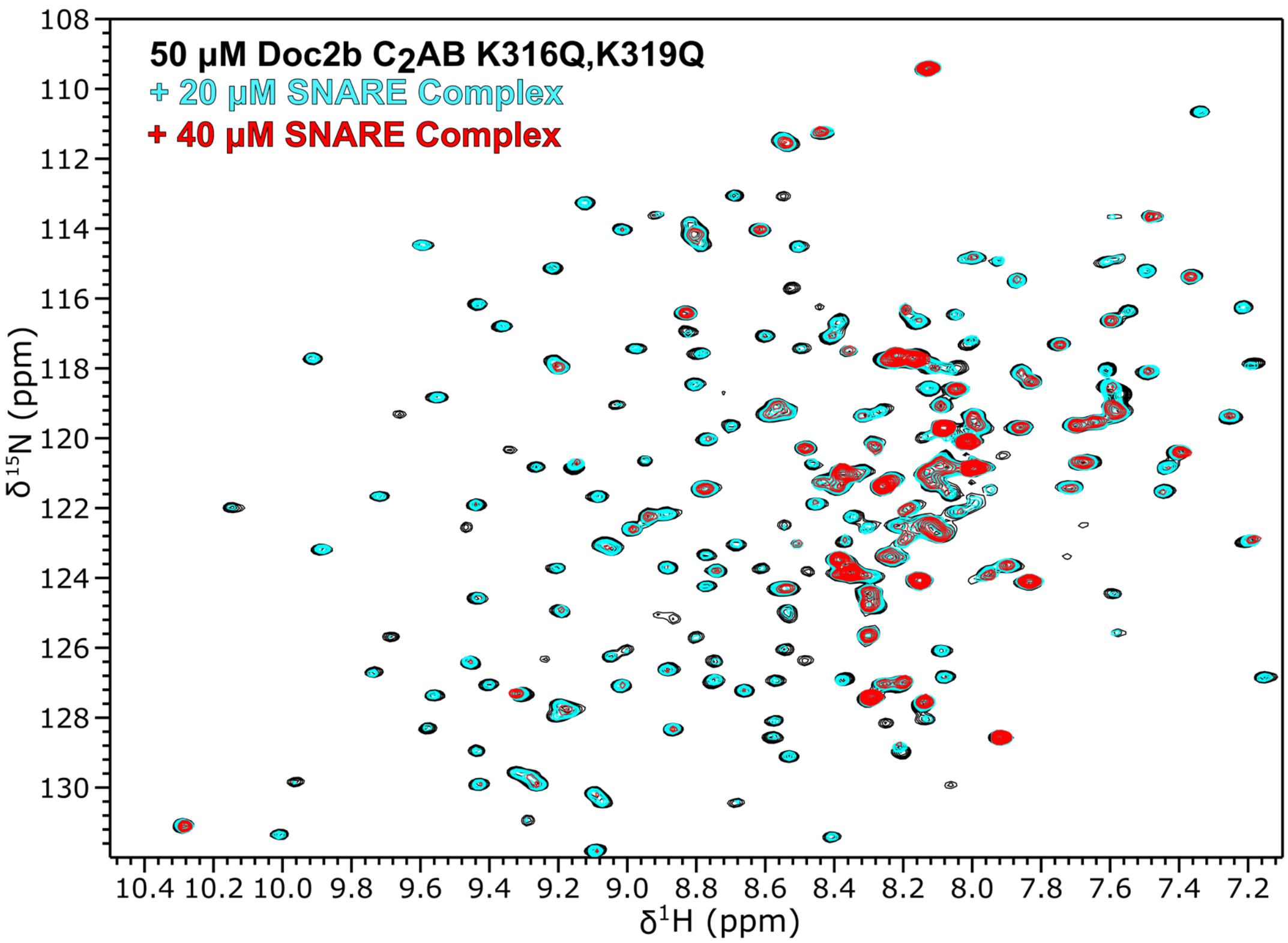

Rastegar et al.
Figure S8

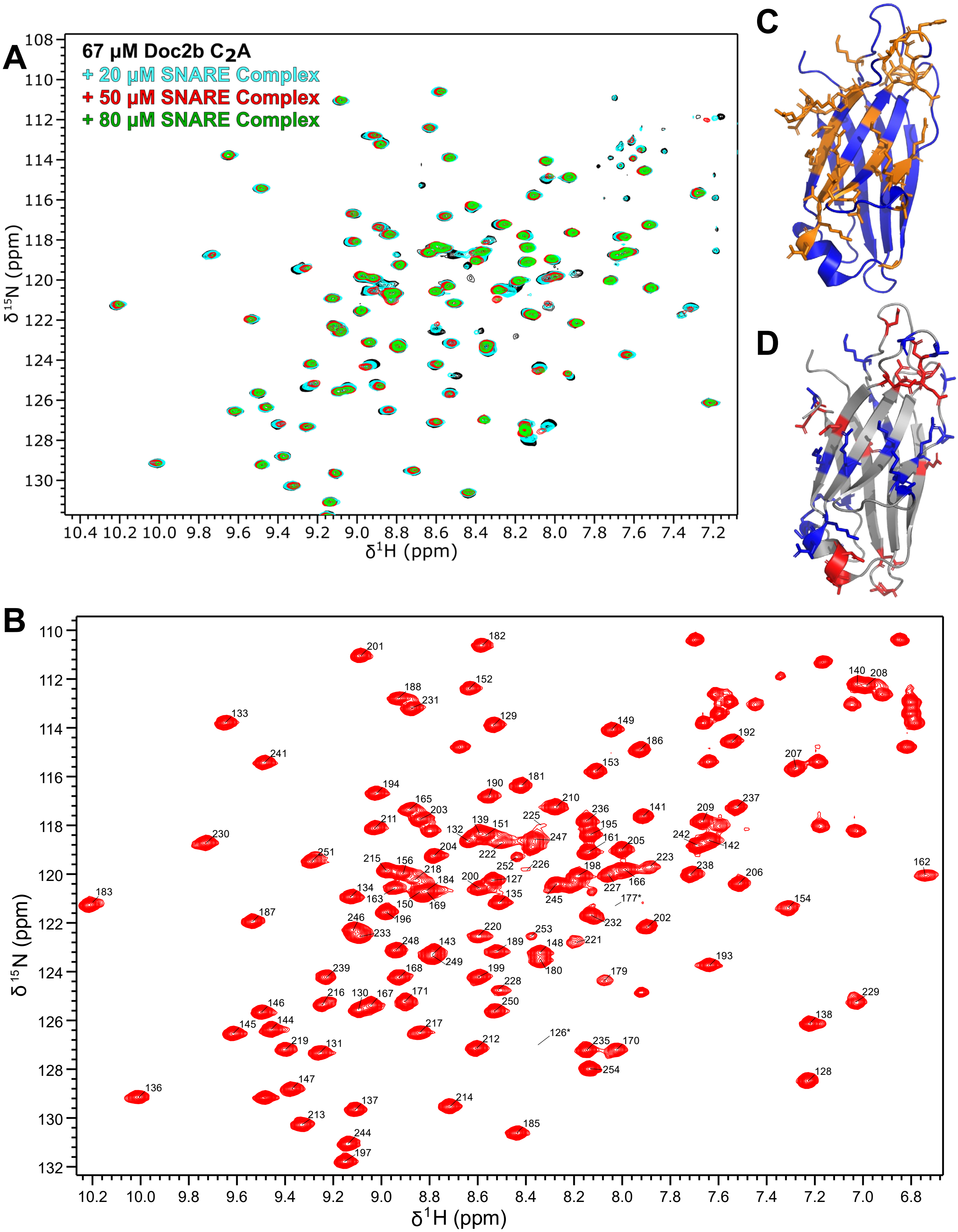

Rastegar et al.
Figure S9

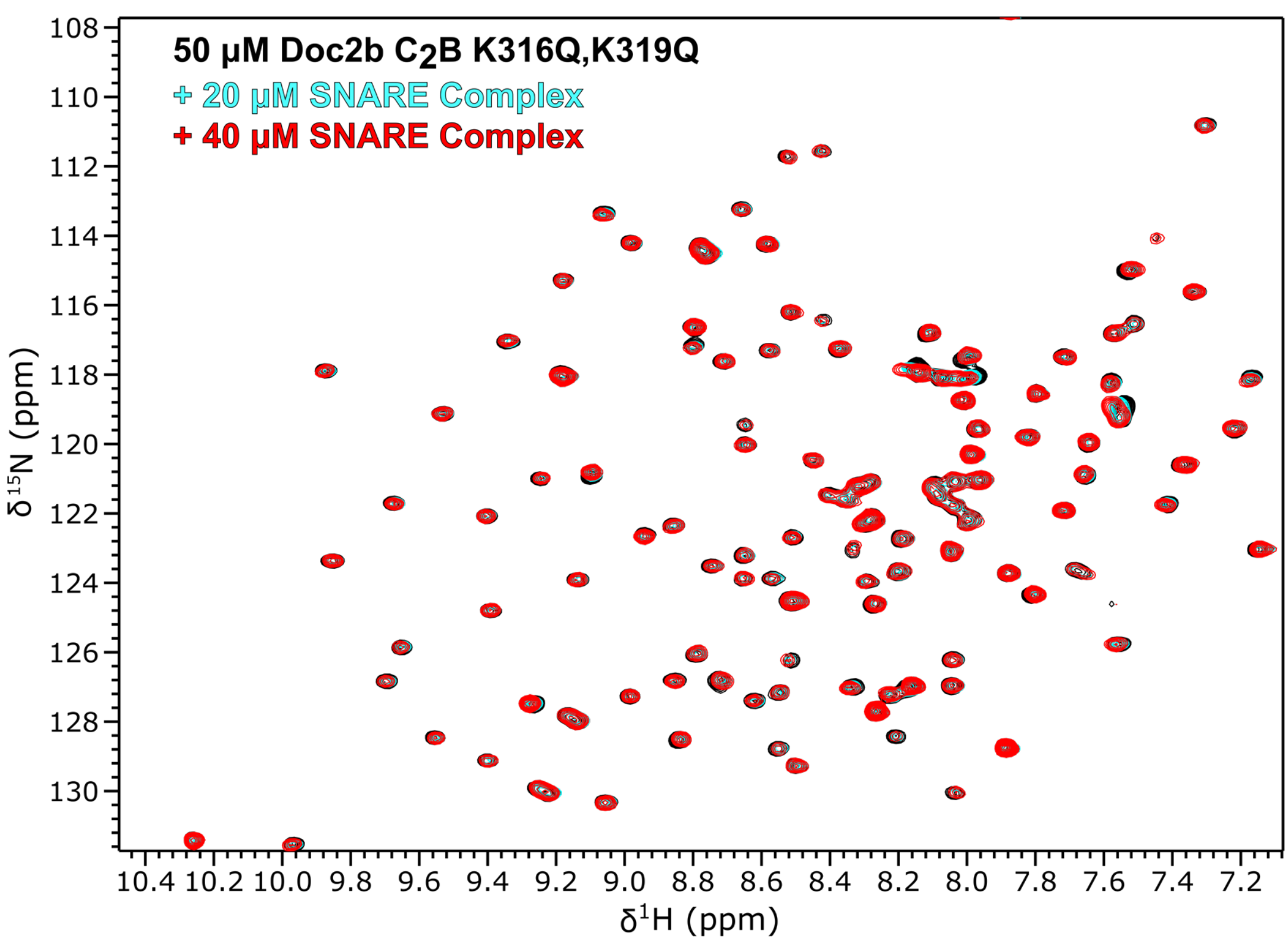

Rastegar et al.
Figure S10

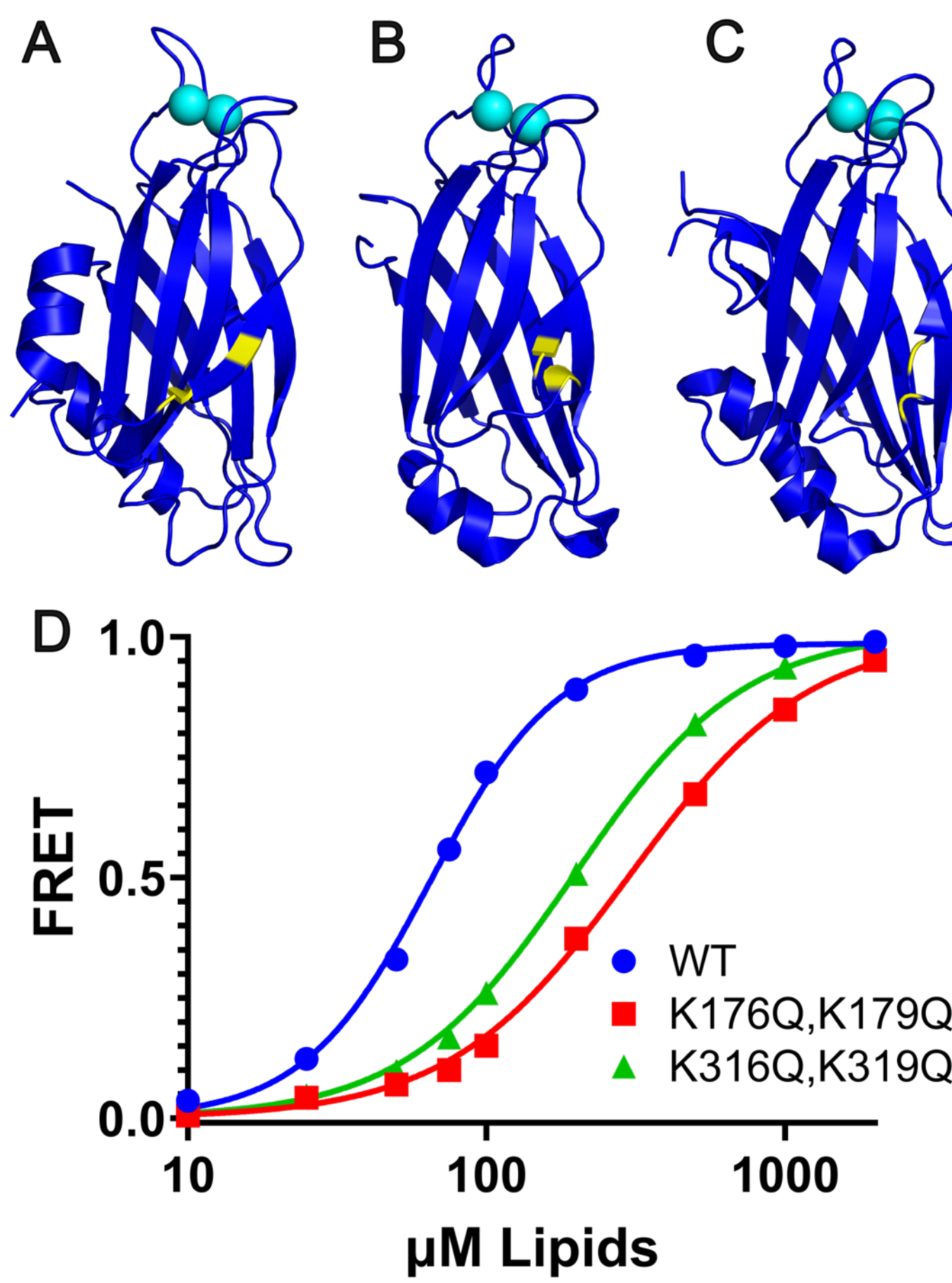

Rastegar et al.
Figure S11

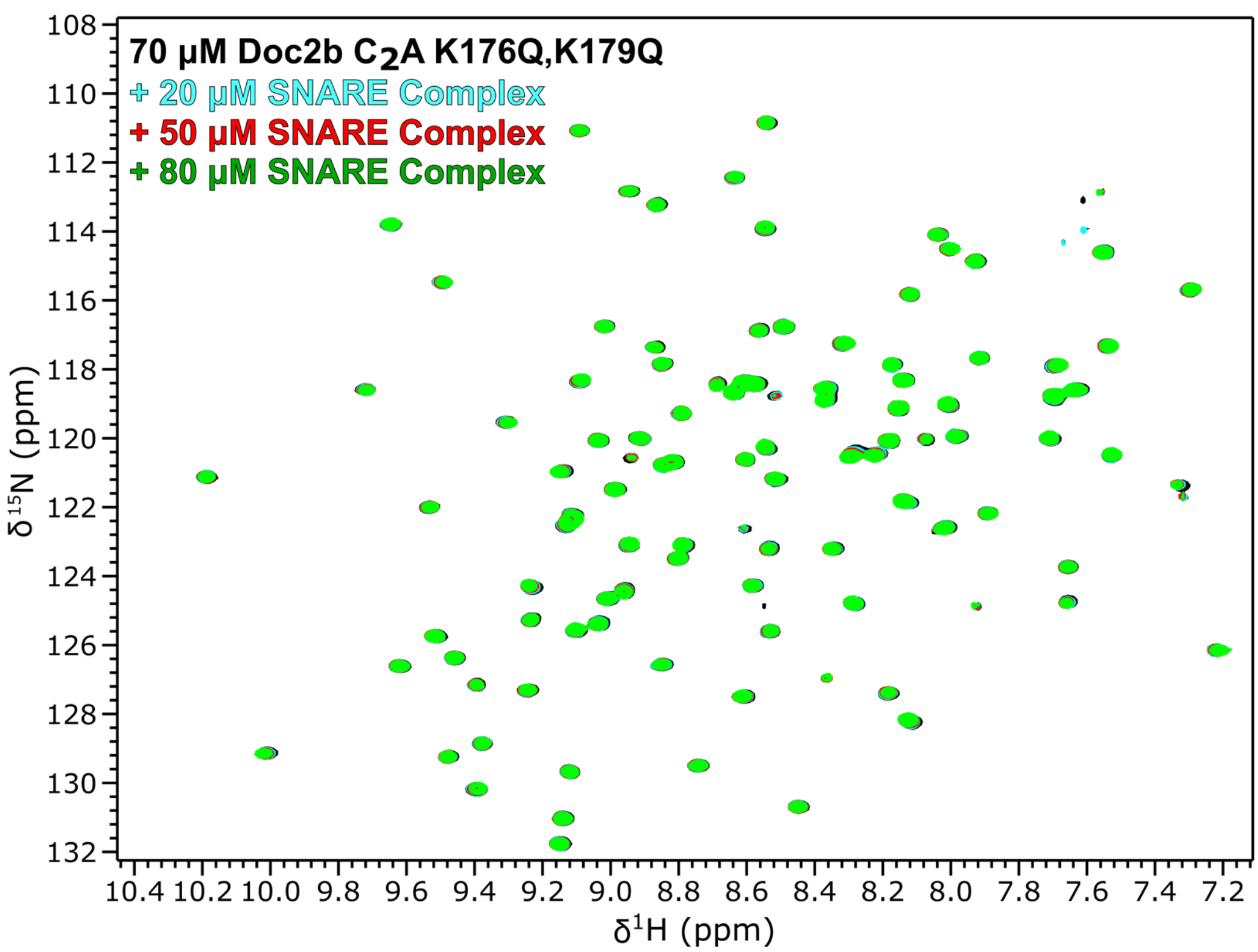

Rastegar et al.
Figure S12

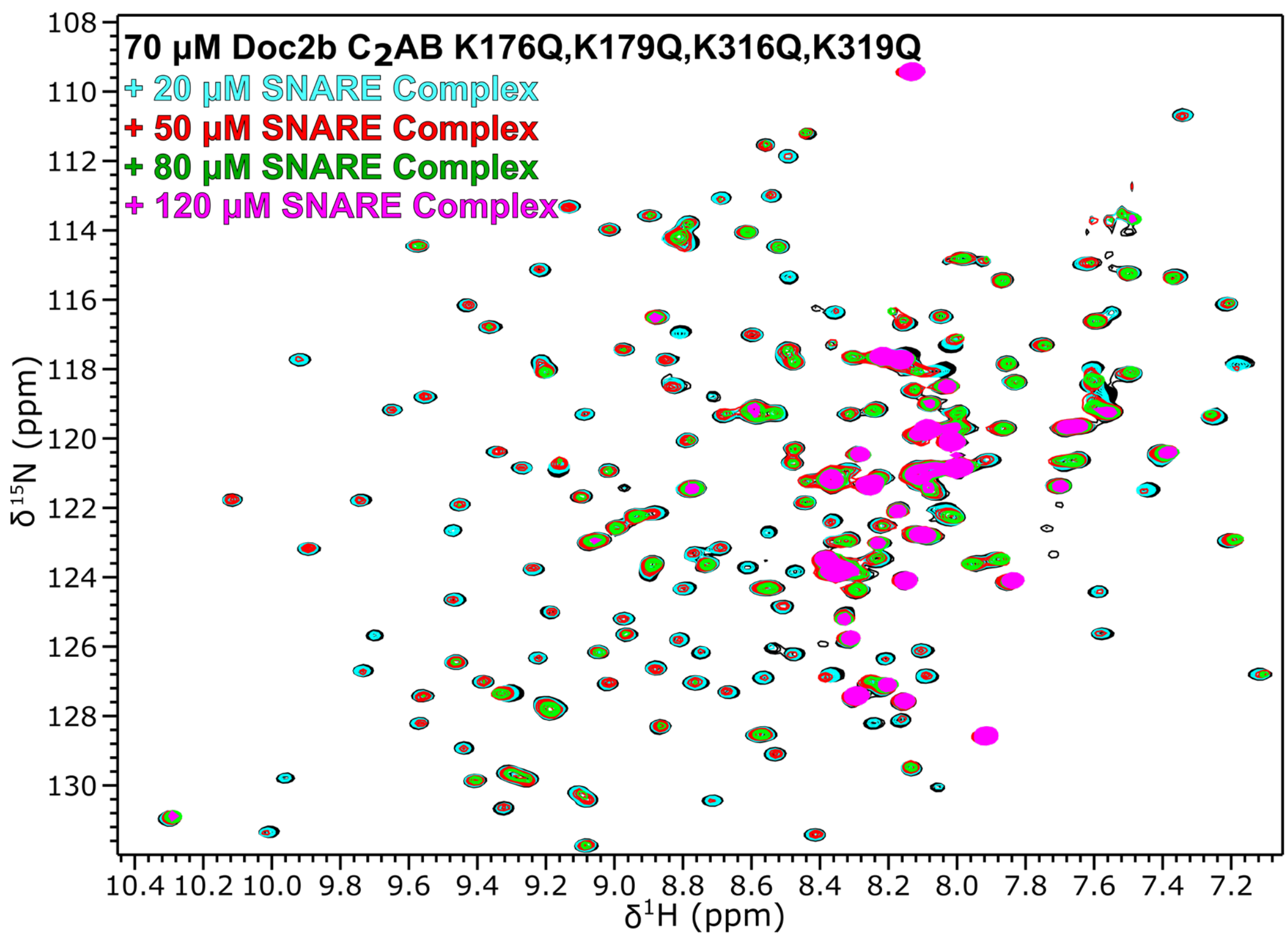

Rastegar et al.
Figure S13

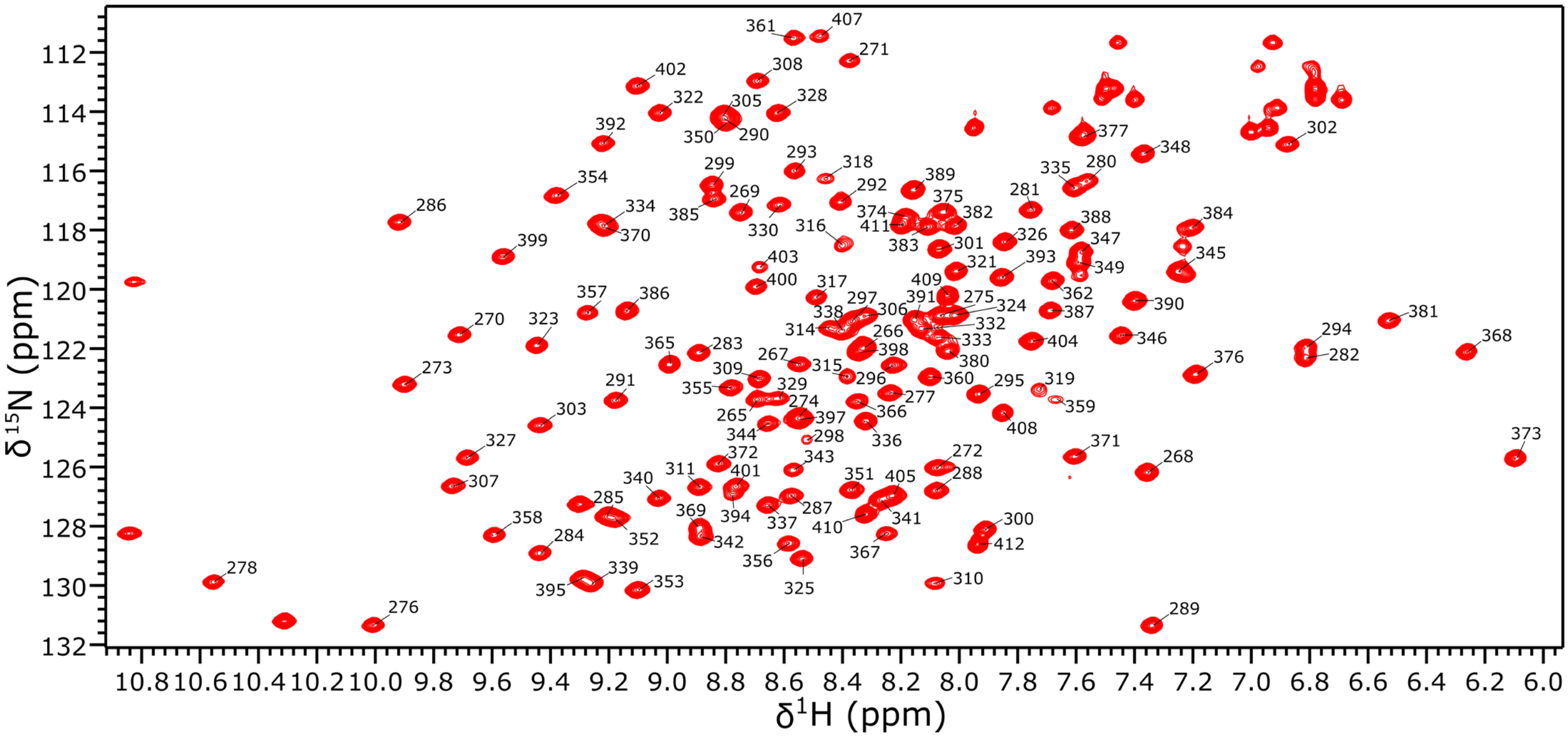

Rastegar et al.
Figure S14

Helix A

					*				*				*	
Rph3A/651-664	K	G	E	R	L	K	H	W	Y	E	C	L	K	N
Doc2B/377-390	K	G	E	R	L	K	H	W	F	D	C	L	K	N
Doc2C/354-367	S	G	D	R	L	R	H	W	C	E	C	L	S	H
Doc2A/365-378	R	G	E	A	Q	K	H	W	R	D	C	L	H	Q
Syt1/383-396	T	G	A	E	L	R	H	W	S	D	M	L	A	N
Syt7/377-390	G	P	G	E	V	K	H	W	K	D	M	I	A	R

Helix B

			*			*	
Rph3A/616-622	K	H	S	D	L	A	K
Doc2B/342-348	K	H	G	D	L	A	K
Doc2C/319-325	L	R	E	E	L	A	Q
Doc2A/330-336	E	L	S	T	L	A	T
Syt1/348-354	P	F	E	Q	I	Q	K
Syt7/342-348	P	T	E	K	L	R	E

Rastegar et al.
Figure S15

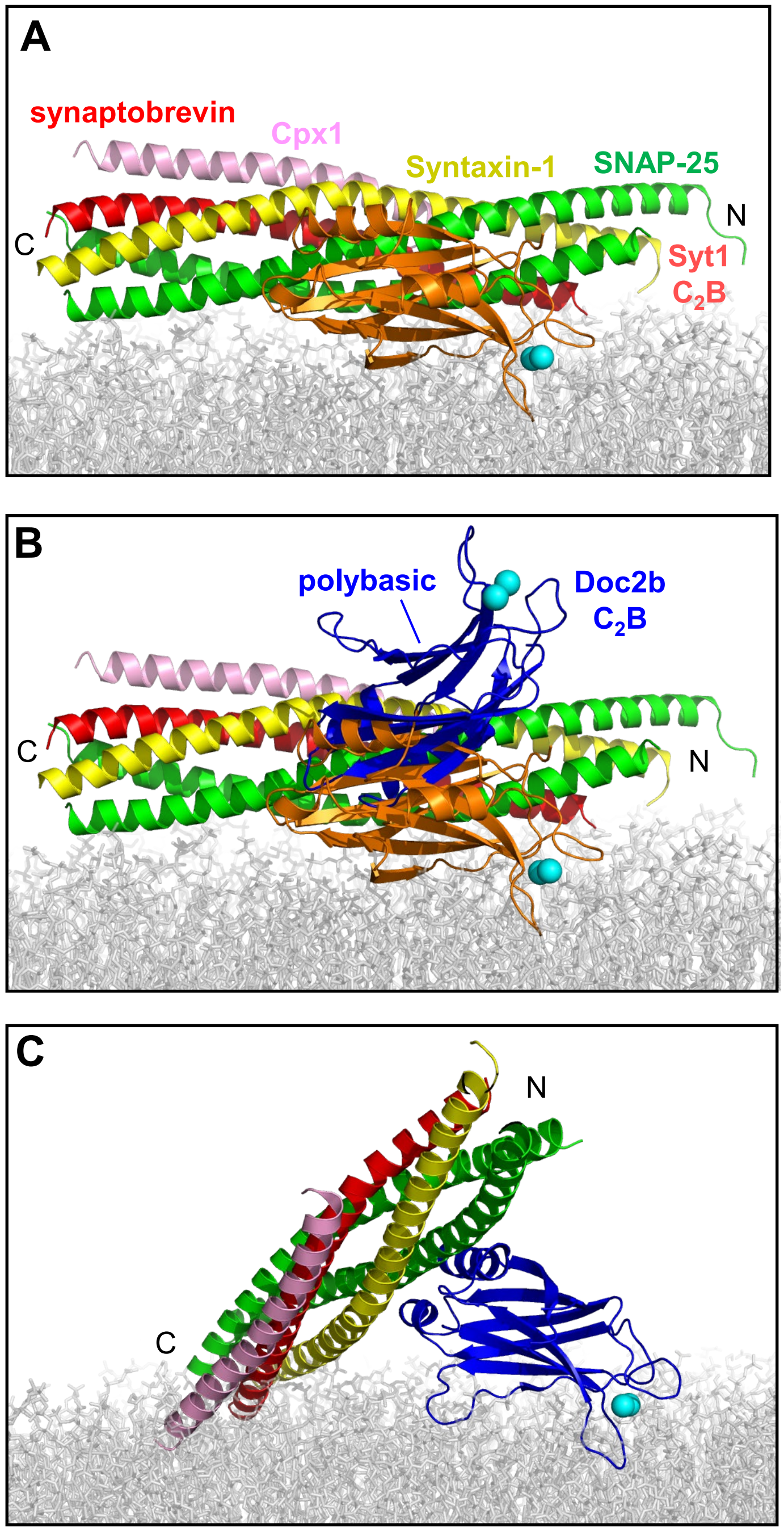

Rastegar et al.
Figure S16

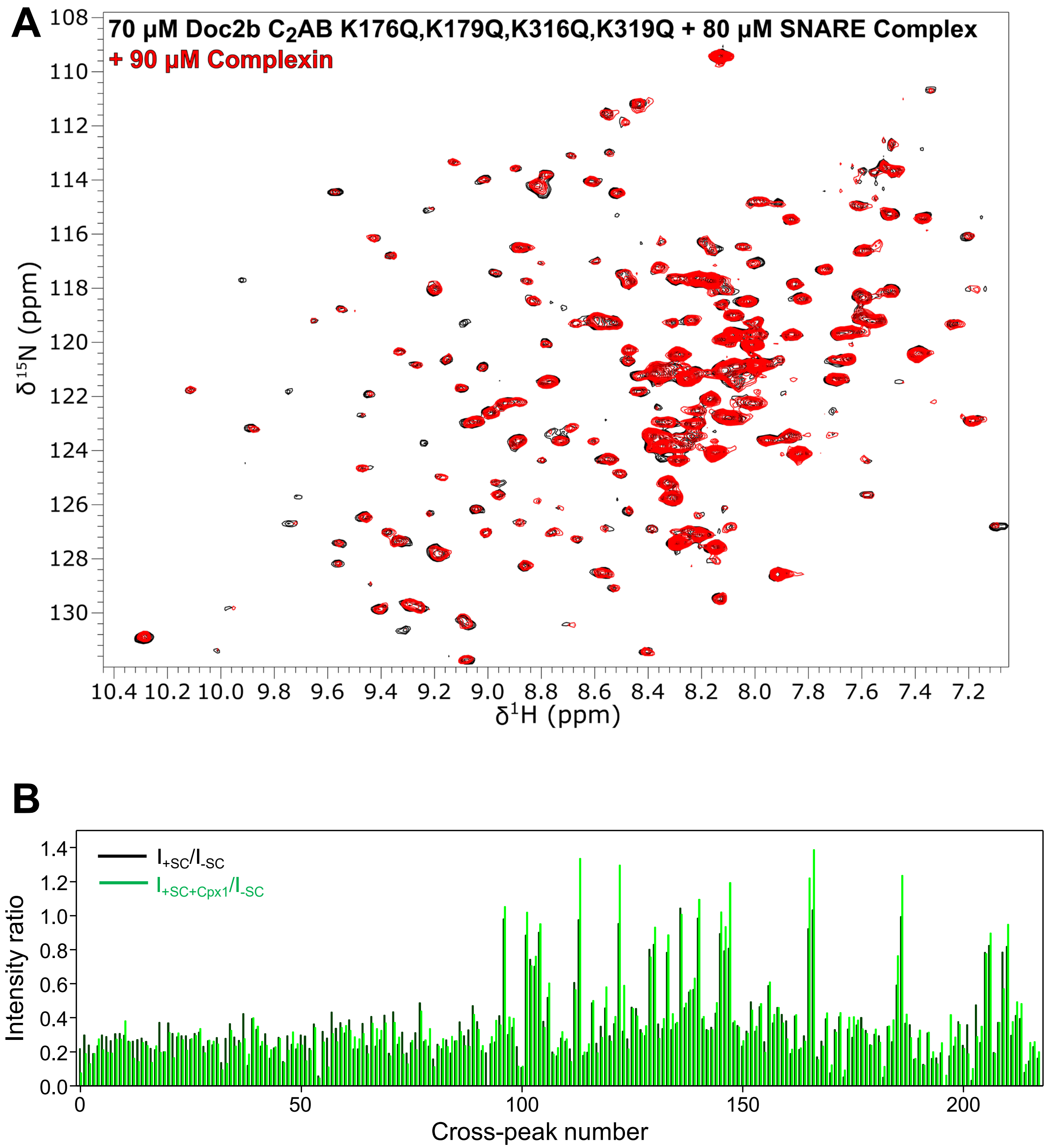

Rastegar et al.
Figure S17

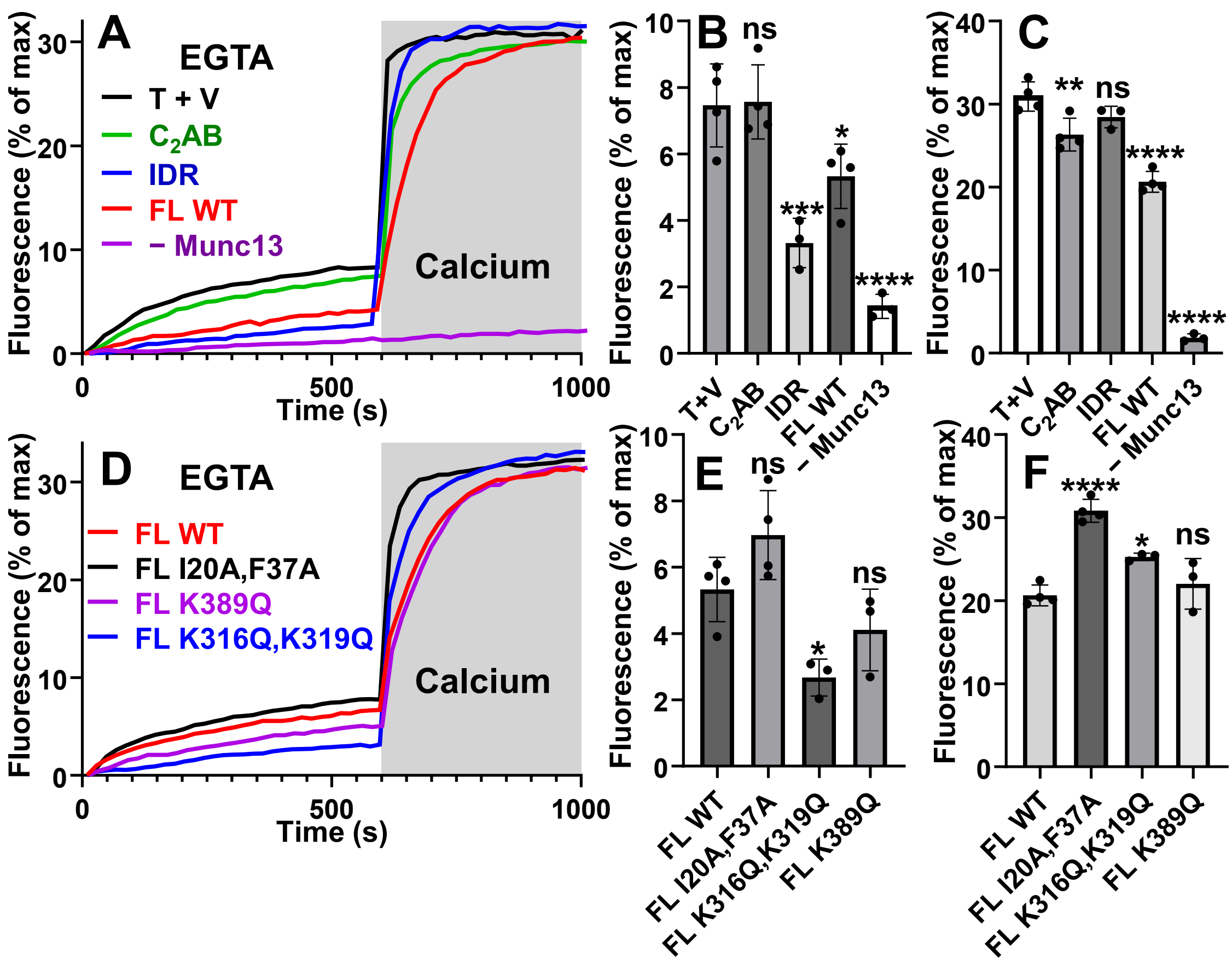

Rastegar et al.
Figure S18

SI References

1. Q. Zhou *et al.*, Architecture of the synaptotagmin-SNARE machinery for neuronal exocytosis. *Nature* **525**, 62-67 (2015).
2. J. Rizo, L. Sari, Y. Qi, W. Im, M. M. Lin, All-atom molecular dynamics simulations of Synaptotagmin-SNARE-complexin complexes bridging a vesicle and a flat lipid bilayer. *Elife* **11**, e76356 (2022).
3. B. Quade *et al.*, Membrane bridging by Munc13-1 is crucial for neurotransmitter release. *Elife* **8**, e42806 (2019).
4. M. Zhao *et al.*, Mechanistic insights into the recycling machine of the SNARE complex. *Nature* **518**, 61-67 (2015).
5. X. Liu *et al.*, Simultaneous lipid and content mixing assays for in vitro reconstitution studies of synaptic vesicle fusion. *Nat Protoc* **12**, 2014-2028 (2017).
6. D. Arac *et al.*, Close membrane-membrane proximity induced by Ca(2+)-dependent multivalent binding of synaptotagmin-1 to phospholipids. *Nat. Struct. Mol. Biol* **13**, 209-217 (2006).
7. X. Chen *et al.*, SNARE-Mediated Lipid Mixing Depends on the Physical State of the Vesicles. *Biophys. J* **90**, 2062-2074 (2006).
8. K. P. Stepien, J. Rizo, Synaptotagmin-1-, Munc18-1-, and Munc13-1-dependent liposome fusion with a few neuronal SNAREs. *Proc Natl Acad Sci U S A* **118** (2021).
9. A. J. Kreutzberger, B. Liang, V. Kiessling, L. K. Tamm, Assembly and Comparison of Plasma Membrane SNARE Acceptor Complexes. *Biophys J* **110**, 2147-2150 (2016).
10. J. Tang *et al.*, A complexin/synaptotagmin 1 switch controls fast synaptic vesicle exocytosis. *Cell* **126**, 1175-1187 (2006).
11. R. Voleti, K. Jaczynska, J. Rizo, Ca(2+)-dependent release of Synaptotagmin-1 from the SNARE complex on phosphatidylinositol 4,5-bisphosphate-containing membranes. *Elife* **9**, e57154 (2020).
12. I. Brouwer *et al.*, Direct quantitative detection of Doc2b-induced hemifusion in optically trapped membranes. *Nat Commun* **6**, 8387 (2015).
13. K. M. Abe, G. Li, Q. He, T. Grant, C. J. Lim, Small LEA proteins mitigate air-water interface damage to fragile cryo-EM samples during plunge freezing. *Nat Commun* **15**, 7705 (2024).
14. M. Pacesa *et al.*, One-shot design of functional protein binders with BindCraft. *Nature* **646**, 483-492 (2025).
15. J. Abramson *et al.*, Accurate structure prediction of biomolecular interactions with AlphaFold 3. *Nature* **630**, 493-500 (2024).
16. S. Keller *et al.*, High-precision isothermal titration calorimetry with automated peak-shape analysis. *Anal Chem* **84**, 5066-5073 (2012).
17. J. C. Houtman *et al.*, Studying multisite binary and ternary protein interactions by global analysis of isothermal titration calorimetry data in SEDPHAT: application to adaptor protein complexes in cell signaling. *Protein Sci* **16**, 30-42 (2007).
18. C. A. Brautigam, Calculations and Publication-Quality Illustrations for Analytical Ultracentrifugation Data. *Method Enzymol* **562**, 109-133 (2015).
19. G. Grynkiewicz, M. Poenie, R. Y. Tsien, A new generation of Ca2+ indicators with greatly improved fluorescence properties. *J Biol Chem* **260**, 3440-3450 (1985).
20. R. Tsien, T. Pozzan, Measurement of cytosolic free Ca2+ with quin2. *Methods Enzymol* **172**, 230-262 (1989).
21. A. Punjani, J. L. Rubinstein, D. J. Fleet, M. A. Brubaker, cryoSPARC: algorithms for rapid unsupervised cryo-EM structure determination. *Nat Methods* **14**, 290-296 (2017).
22. P. B. Rosenthal, R. Henderson, Optimal determination of particle orientation, absolute hand, and contrast loss in single-particle electron cryomicroscopy. *J Mol Biol* **333**, 721-745 (2003).

23. E. C. Meng *et al.*, UCSF ChimeraX: Tools for structure building and analysis. *Protein Sci* **32**, e4792 (2023).
24. K. Kim, H. Li, O. B. Clarke, High-resolution ab initio reconstruction enables cryo-EM structure determination of small particles, *bioRxiv*, doi: https://doi.org/10.1101/2025.09.08.674935 (2025).
25. T. Bepler *et al.*, Positive-unlabeled convolutional neural networks for particle picking in cryo-electron micrographs. *Nat Methods* **16**, 1153-1160 (2019).
26. H. Cao *et al.*, EMReady2: improvement of cryo-EM and cryo-ET maps by local quality-aware deep learning with Mamba. *Nat Commun* **17,** 6493 (2026).
27. P. D. Adams *et al.*, PHENIX: a comprehensive Python-based system for macromolecular structure solution. *Acta Crystallogr. D. Biol. Crystallogr* **66**, 213-221 (2010).
28. T. I. Croll, ISOLDE: a physically realistic environment for model building into low-resolution electron-density maps. *Acta Crystallogr D Struct Biol* **74**, 519-530 (2018).
29. P. Emsley, B. Lohkamp, W. G. Scott, K. Cowtan, Features and development of Coot. *Acta Crystallogr. D. Biol. Crystallogr* **66**, 486-501 (2010).
30. F. Madeira *et al.*, The EMBL-EBI Job Dispatcher sequence analysis tools framework in 2024. *Nucleic Acids Res* **52**, W521-W525 (2024).
31. A. M. Waterhouse, J. B. Procter, D. M. Martin, M. Clamp, G. J. Barton, Jalview Version 2--a multiple sequence alignment editor and analysis workbench. *Bioinformatics* **25**, 1189-1191 (2009).
32. K. D. Brewer *et al.*, Dynamic binding mode of a Synaptotagmin-1-SNARE complex in solution. *Nat. Struct. Mol. Biol* **22**, 555-564 (2015).
33. C. Ferrer-Orta *et al.*, Structural characterization of the Rabphilin-3A-SNAP25 interaction. *Proc Natl Acad Sci U S A* **114**, E5343-E5351 (2017).
34. X. Chen *et al.*, Three-dimensional structure of the complexin/SNARE complex. *Neuron* **33**, 397-409 (2002).
